# Liberation mathematics I: the behavioral and consciousness definition of perpetual self-transcendence

**DOI:** 10.3389/fpsyg.2026.1741096

**Published:** 2026-05-04

**Authors:** Swapan Samanta

**Affiliations:** Indoriv Clinical Research Centre, Kolkata, India

**Keywords:** behavioral mathematics, consciousness, empirical spirituality, liberation, moksha, phenomenology, self-transcendence

## Abstract

Traditional definitions of liberation (such as moksha, nirvana, and kaivalya) across Hindu, Buddhist, and Jain philosophical traditions have remained empirically underspecified and conceptually divergent for over 2,500 years. This study proposes an operational definition of liberation asperpetual self-transcendence through infinite domain expansion, characterized by the inability to fix one’s identity in any achieved position. The framework employs mathematical formalization of behavioral observables, comparative analysis across six traditions, and empirically testable criteria, including the Liberation Coefficient (LC) and Self-Transcendence Rate (STR). The model resolves five traditional controversies by demonstrating liberation as: (1) process rather than state, (2) ego fluidity rather than dissolution, (3) mandatory world engagement, (4) simultaneous requirement of all paths, and (5) both sudden recognition and continuous application. This framework may provide a foundation for systematic empirical investigation of consciousness liberation, establishing Liberation Science (Liberology) as a measurable behavioral and consciousness phenomenon.

## 1 Introduction

### 1.1 The problem of defining liberation

The concept of liberation represents the ultimate aim of human existence across multiple philosophical traditions (Eliade, 1969; Radhakrishnan, 1923; Zimmer, 1951). However, despite millennia of contemplative practice, liberation remains empirically underspecified and conceptually divergent. Traditional formulations describe liberation as: (a) realization of atman–Brahman identity in Advaita Vedanta (Deutsch, 1969; Shankara, 2023), (b) separation of Purusha from Prakriti in Yoga philosophy (Patanjali, 2009; Feuerstein, 2001), (c) cessation of suffering in Buddhism (Bodhi, 2000; Rahula, 1974), (d) loving union with God in Dvaita Vedanta (Sharma, 2000), and (e) karmic purification in Jainism (Jaini, 1979; Tatia, 1994). These formulations lack operational definitions that can be subjected to empirical investigation.

Contemporary consciousness studies (Velmans and Schneider, 2007; Zelazo et al., 2007), contemplative neuroscience (Davidson and Lutz, 2008; Tang et al., 2015), predictive processing frameworks (Clark, 2013; Friston, 2010), and metacognitive research (Barrett et al., 2024) create opportunities for reformulating liberation in empirically tractable terms. This approach differs from existing contemplative science programs by providing mathematical formalization of behavioral observables rather than focusing solely on subjective meditation experiences.

### 1.2 Unresolved controversies

**Figure 1:**
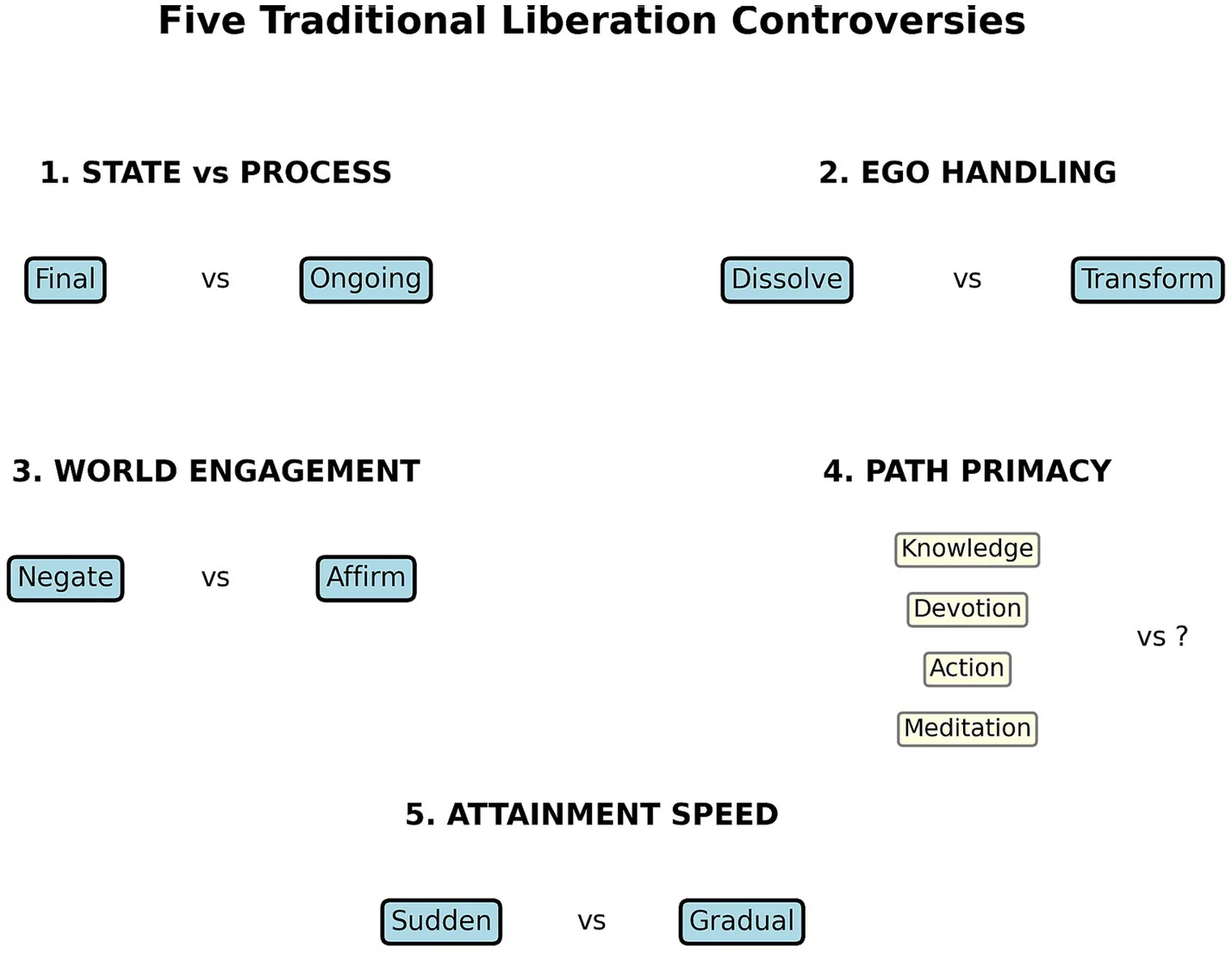
Schematic representation of five unresolved controversies in liberation philosophy across traditional systems.

Five major controversies persist across traditions (Figure 1):

*Controversy 1: State versus process*. Whether liberation represents a terminal state or an ongoing developmental process (Griffiths, 1986; Forman, 1999).

*Controversy 2: ego dissolution versus transformation*. Whether the individual self must be eliminated or transformed (MacKenzie, 2008; Thompson, 2015).

*Controversy 3: world negation versus affirmation*. Whether liberation requires withdrawal or active participation (Bhattacharya, 1943; Chakrabarti, 1983).

*Controversy 4: path primacy*. Whether knowledge (jnana), devotion (bhakti), action (karma), or meditation (dhyana) represents the primary path (Minor, 1986; Lipner, 1997).

*Controversy 5: Sudden versus gradual attainment*. Whether liberation occurs instantaneously or requires extended progression (Hori, 1994; Sharf, 1995).

### 1.3 Objectives

This study presents a novel operational definition of liberation that facilitates: (a) cross-cultural comparative analysis, (b) systematic investigation of developmental trajectories, (c) identification of behavioral and neurophysiological markers, (d) assessment of intervention effectiveness, and (e) integration with contemporary cognitive sciences. The specific objectives include:

1. Formalizing liberation mathematically using behavioral observables.
2. Demonstrating the resolution of five traditional controversies.
3. Developing empirically testable criteria and measurement protocols.
4. Comparing the proposed model with six major philosophical traditions.
5. Establishing a foundation for systematic empirical investigation.

### 1.4 Methodology

Throughout this study, philosophical traditions and their foundational texts are not cited merely as authorities but as sources of conceptual insights, subsequently reformulated into operational terms. When referencing Shankara, Patanjali, the Buddha, Husserl, or Heidegger, this study extracts their core insights on consciousness transformation and translates into the mathematical framework’s variables and conditions. This approach respects traditional wisdom while subjecting it to the requirements of empirical tractability. The arguments contained in the referenced works are made explicit within the text itself, demonstrating how traditional concepts map onto the proposed formalization.

#### 1.4.1 Section 1 summary: the problem and the response

*This section has identified a 2,500-year definitional impasse: liberation (moksha, nirvana, and kaivalya) remains empirically under-specified across traditions, generating five persistent controversies that resist resolution within traditional frameworks. Each tradition captures genuine aspects of liberation, yet none provides criteria amenable to systematic investigation. In response, this study proposes an operational definition—liberation as perpetual self-transcendence, characterized by the inability to fix identity in any achieved position. The following section develops this definition into a formal mathematical framework with measurable behavioral correlates, translating philosophical intuitions into testable hypotheses.*

## 2 Theoretical framework

### 2.1 Core definition

Liberation (L) is operationally defined as a dynamic state of consciousness characterized by perpetual self-transcendence through infinite domain expansion, wherein identity positioning remains fluid and resists solidification around any achieved state, maintained through continuous application of behavioral and cognitive patterns that prevent identity fixation.

### 2.2 Mathematical formalization

The liberation function L(*t*) at time *t* is formally defined as:

L(*t*) = True ⟺ All conditions simultaneously satisfied:

The conditions include:

1. *dE*/*dt* >0 (evolution rate positive),
2. A(*t*) < Asp(*t* + Δt) ∀t (achievement < aspiration),
3. PF(*t*) → 0 (position fixation → zero),
4. TD(*t*) → ∞ (testing domains → infinity),
5. FP(*t*) → ∞ ∧ EC(*t*) = retained (fresh perception ∧ expert capability),

where

- E(*t*) = Evolutionary development at time *t*,
- A(*t*) = Achievement level at time *t*,
- Asp(*t*) = Aspiration level at time *t*,
- PF(*t*) = Position fixation (identity solidification),
- TD(*t*) = Number of testing domains encountered,
- FP(*t*) = Fresh perception coefficient, and
- EC(*t*) = Expert capability retention.

Interpretive note: These five conditions operationalize liberation as a dynamic configuration rather than a static achievement. Condition 1 (*dE*/*dt* > 0) ensures consciousness never ‘arrives’—a liberated individual who declares completion has, by this definition, become imprisoned in the identity of ‘one who has completed.’ Condition 2 (A < Asp) formalizes perpetual aspiration exceeding achievement; the moment satisfaction equals accomplishment, identity crystallizes around that accomplishment. Condition 3 (PF → 0) captures the resistance to solidify around any position, including spiritual ones—even ‘enlightenment’ cannot become a resting place. Condition 4 (TD → ∞) requires continuous entry into unfamiliar testing domains, because liberation cannot be demonstrated solely within the individual’s expertise, where identity pattern may remain unchallenged. Condition 5 paradoxically demands that expert capability (EC) remain intact while fresh perception (FP) approaches the unlimited—the master retains mastery yet perceives with beginner’s eyes.

**Figure 2:**
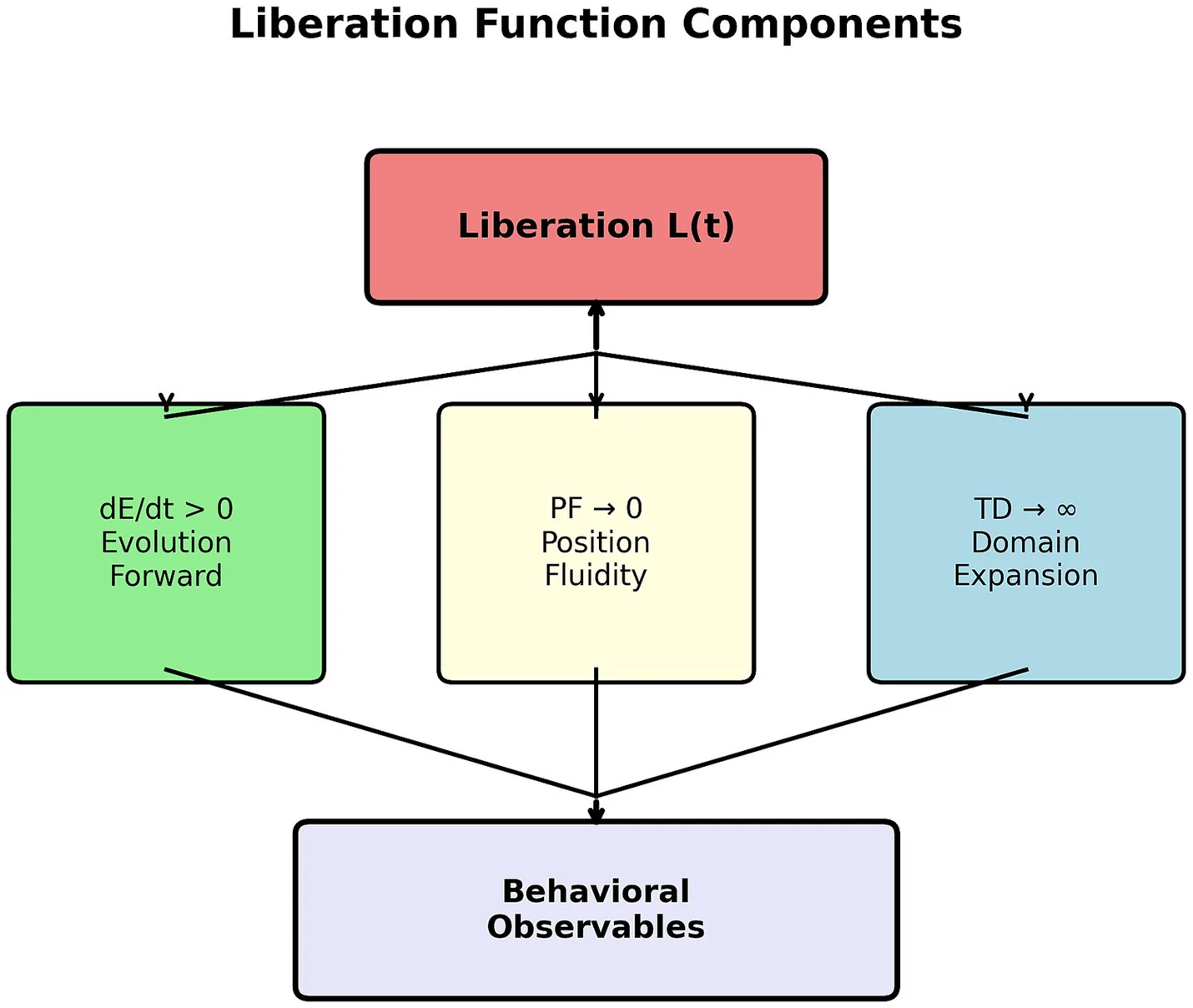
Causal relationships between core liberation components, showing how evolution rate, position fluidity, and domain expansion contribute to observable liberation behaviors.

In simpler terms: Liberation requires simultaneously maintaining forward momentum (*dE*/*dt* > 0), never satisfying aspirations with current achievements, avoiding identity fixation around any position, continuously entering new testing domains, and paradoxically combining expert-level skills with beginner-level perceptual freshness (Figure 2).

### 2.3 Observable behavioral criteria

Liberation manifests through five empirically observable patterns, namely:

*Criterion 1: Non-Identification with achievement*. Achievement immediately catalyzes pursuit of greater challenges rather than identity consolidation.

*Criterion 2: Active position negation*. Systematic engagement in behaviors undermining one’s own authority positions (e.g., a teacher creating superior students).

*Criterion 3: Continuous domain expansion*. Active seeking of untested domains, including those outside formal expertise.

*Criterion 4: Expert–beginner paradox*. Simultaneous maintenance of expert capability with beginner-level perceptual freshness.

*Criterion 5: No terminus identification*. Inability to claim final attainment; recognition that identifying with the ‘enlightened’ state itself constitutes imprisonment.

### 2.4 Key measurement functions

Self-Transcendence Rate (STR):

$\begin{array}{l} \mathrm{STR}(t)=[d(\mathrm{New}\_\text{Domains}\_\text{Mastered})/\mathrm{dt}]\\ \times [d(\text{Past}\_\text{Identities}\_\text{Released})/\mathrm{dt}] \end{array}$

Liberation Coefficient (LC):

$\begin{array}{l} \mathrm{LC}(t)=\sum (\mathrm{New}\_\text{Testing}\_\text{Domains}\_\text{Entered})\\ /\sum (\text{Past}\_\text{Achievements}\_\text{Referenced}) \end{array}$

Interpretation:

- LC → ∞: liberated (continuous domain entry),
- LC → 0: imprisoned (fixated on past achievement).
- LC ≈ 1: stagnant (balanced but not advancing).

Concrete example: Consider a renowned physicist who, after receiving a Nobel Prize, (a) begins studying classical music composition, (b) volunteers to teach science to underprivileged children, (c) enters psychotherapy to examine personal blind spots, and (d) mentions the Nobel Prize only once in 6 months when directly asked. In this case, new domains entered = 3; past achievement references = 1; and LC = 3.0, indicating high liberation. When compared with another physicist who (a) gives frequent interviews emphasizing the prize, (b) declines invitations to speak outside physics, and (c) references the award in unrelated professional contexts, new domains = 0; references = 15; and LC = 0, indicating complete imprisonment. The contrast illustrates the operational distinction between liberation and imprisonment.

Beginner–expert paradox function (BEP):

BEP(t)=[EC(t)×FP(t)]/IF(t)

where EC(*t*) is the expert capability, FP(*t*) is the fresh perception, and IF(*t*) is the identity fixation. Furthermore, when IF(*t*) → 0 and FP(*t*) → ∞, then BEP(*t*) → ∞ (liberation).

### 2.5 Critical distinction: liberation versus pathological fixation

A crucial clarification prevents misinterpretation: genuine liberation must be distinguished from its pathological mimic—rigid identification with singular spiritual forms.

#### 2.5.1 The pathological mimic

Clinical presentation: Individuals rigidly identify with a single spiritual concept (‘I AM the ocean’), potentially leading to self-destructive behaviors (attempting to merge with the ocean through drowning or jumping into fire).

Mathematical signature:

The mathematical signature defines liberation as,

L_path(t)=dN_id/dt→0

where N_id is the number of distinct identities adopted. The result may be interpreted as zero identity fluidity (L_path → 0).

Psychiatric classification: Severe identity fixation indicating psychotic identification (grandiose delusions), dissociative disorder, acute manic episode, or borderline personality organization.

Critical distinguisher: Inability to shift perspective voluntarily.

#### 2.5.2 Genuine liberation

Authentic presentation: Infinite identity fluidity—capacity to perceive consciousness in all phenomena and fluidly adopt any perspective without fixation, maintaining meta-stability within dynamic integration networks.

Mathematical signature:

The mathematical signature defines liberation as,

$$\begin{array}{l} L\_\mathrm{lib}(t)=\mathrm{dN}\_\mathrm{id}/\mathrm{dt}\to \text{High finite value}\\ \;(50–200\;\text{perspectives}/\mathrm{day}) \end{array}$$

Neurocognitive basis: From contemporary neuroscience and the Integrated Information Theory (Tononi, 2016; Seth, 2021), liberation corresponds to maximal metacognitive flexibility—the ability to shift between neural self-models while maintaining integrative coherence and reality testing.

Critical distinguisher: Volitional control over identity adoption. A liberated individual can shift perspectives rapidly while maintaining coherence, meet biological needs, maintain social functioning, return to baseline identity when contextually appropriate, and distinguish fluidly adopted perspectives from delusional beliefs.

**Figure 3:**
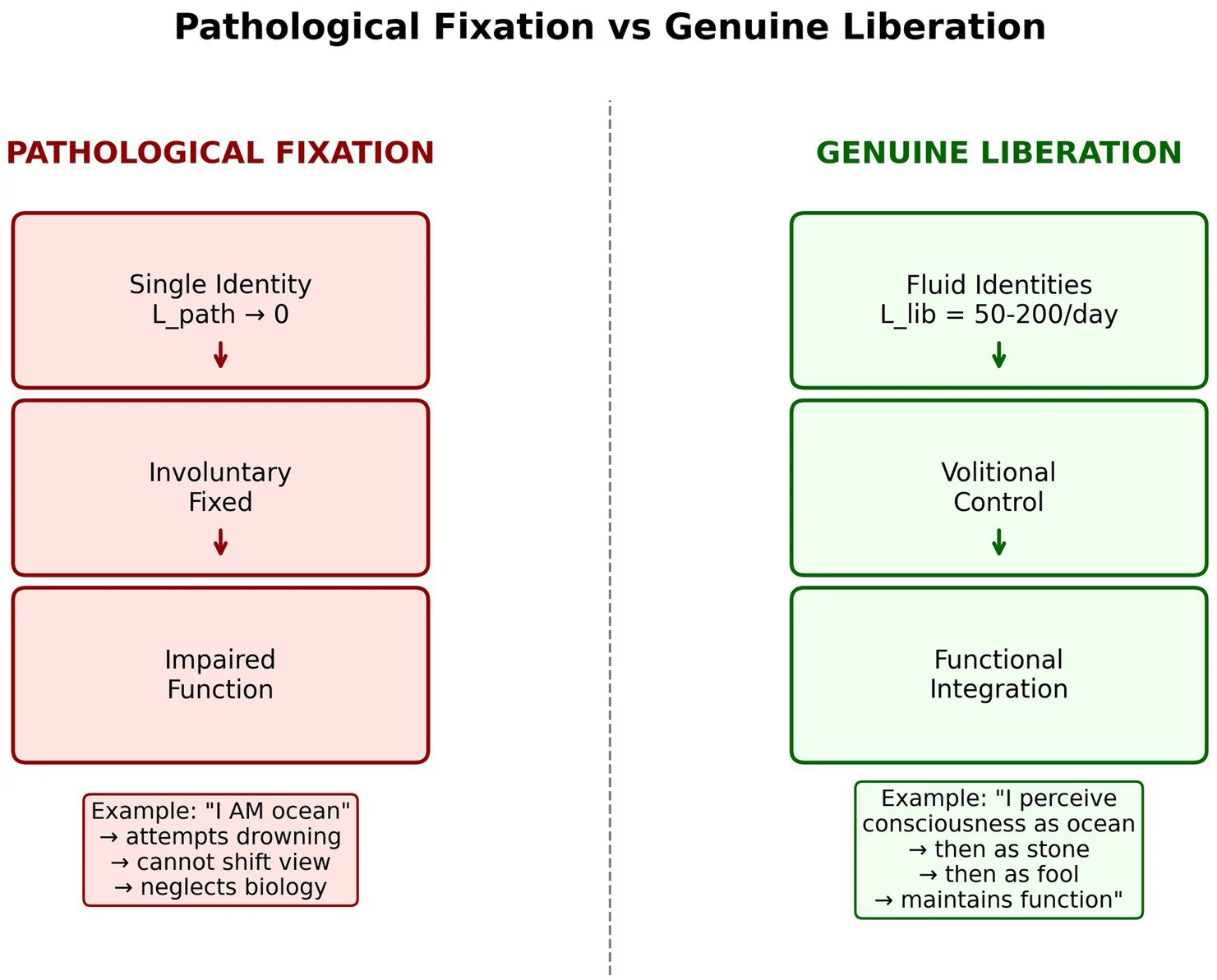
Distinguishing pathological fixation (rigid single identity, L → 0) from genuine liberation (fluid multiple perspectives, L = 50–200/day) under diagnostic criteria.

Clinical illustration: Patient A reports that *‘I am the ocean—there is no separate self*.’ When asked to describe herself from the perspective of a child, she cannot shift, repeating ‘*I am the ocean*.’ When asked about meals, she dismisses biological needs as an illusion. Diagnosis: Pathological fixation (possible psychotic identification; LC approaching 0). Patient B reports the same phrase but, when prompted, fluidly describes herself as a child, then a stone, then a fool, and then returns to ordinary conversation about dinner plans. This is liberation—identity fluidity with preserved functionality (LC > 2.0) (Figure 3).

#### 2.5.3 Clinical assessment protocol

When evaluating claims of spiritual realization, the following tests are performed, namely:

1. Identity fluidity test: ‘Describe yourself from the perspective of: child, elder, enemy, stone, and void.’ Liberated individuals shift fluidly while maintaining coherence, whereas pathological fixation fails to shift and retains a single identity.
2. Functional integration test: ‘How do you meet biological needs?’ Liberated individuals integrate perspectives with functionality, while pathological fixation denies or neglects biological/social needs.
3. Volitional control test: ‘Return to ordinary perspective now.’ Liberated individuals shift immediately, while pathological fixation fails to return and remains fixed in an altered state.
4. Temporal Stability Test: Identity adoption rate over 7 days. Liberated individuals exhibit L(*t*) > 20 perspectives/day, while pathological fixation exhibit L(t) ≈ 0–2 perspectives/day.
5. Reality testing: ‘Is your body real? Do consequences exist?’ Liberated individuals maintain dual awareness (emptiness AND form), while pathological fixation denies physical reality.

Clinical caveat: LC < 0.2 may correlate with dissociative rigidity and warrants careful clinical evaluation before participation in research.

#### 2.5.4 Section 2 summary: from intuition to formal model

*This section has translated the intuitive concept of liberation into a formal framework with five simultaneously required conditions, measurable coefficients (such as LC, STR, and BEP), and a critical distinction between genuine liberation and pathological fixation. The key insight is that liberation is not a state to be achieved but a dynamic process that prevents identity-crystallization—the liberated individual cannot stop transcending because any terminus would itself become a prison. Unlike traditional formulations that describe liberation as realization or attainment, Liberation Mathematics defines it as ongoing inability to fixate. The mathematical formalization enables operationalization: liberation becomes measurable through behavioral observation rather than remaining dependent on subjective self-report or authority validation. The following section examines how this formalization relates to six traditional philosophical frameworks, demonstrating both convergence with traditional insights and resolution of their operational limitations.*

## 3 Comparative analysis: traditional definitions

### 3.1 Advaita Vedanta (non-dualism)

Traditional formulation: Liberation (moksha) represents the realization that atman is identical with Brahman (Deutsch, 1969; Shankara, 2023).

Mathematical expression:

The mathematical expression defines liberation in Advaita Vedanta as,

L_Advaita=lim(avidya→0)[atman≡Brahman]

Key claims:

1. Ego dissolves as a false construct
2. World (maya) is an illusion to be transcended
3. Liberation is a static realization state
4. Knowledge (jnana) is the primary path

#### 3.1.1 Theoretical foundation

Shankara’s analysis of avidyā (ignorance) as adhyāsa (superimposition) illuminates how consciousness mistakenly identifies with limited objects—body, mind, and social role—just as one might mistake a rope for a snake under dim light. The jīva (individual soul) is not ontologically separate from Brahman but merely Brahman under the spell of beginningless ignorance. The mahāvākyas (‘Tat tvam asi’—'That thou art’; ‘Aham Brahmasmi’—'I am Brahman’) function not as metaphysical assertions to be believed but as identity-dissolving recognitions that shatter the superimposed boundaries between the self and the absolute. According to Liberation Mathematics, this corresponds to PF → 0: the removal of superimposed identities reveals consciousness as inherently unfixed. However, Advaita’s emphasis on a terminal state of realization diverges from Liberation Mathematics’ insistence on perpetual process.

#### 3.1.2 Operational problems

The operational problems include the following questions:

- How to empirically verify the atman–Brahman identity?
- If ego dissolves, what sustains biological functioning?
- How to distinguish genuine realization from belief?

### 3.2 Patanjali’s yoga (classical dualism)

Traditional formulation: Liberation (kaivalya) through complete separation of Purusha from Prakriti (Patanjali, 2009; Feuerstein, 2001).

#### 3.2.1 Mathematical expression

The mathematical expression defines liberation in Patanjali’s yoga as,

L_Yoga=∣Purusha–Prakriti∣→∞

Key claims:

1. Consciousness and matter are ontologically distinct;
2. Liberation requires withdrawal from material engagement.
3. Eight-staged developmental process.
4. Final state is characterized by isolation.

Theoretical foundation: Patanjali defines yoga as citta-vṛtti-nirodha—cessation of mental fluctuations—revealing pure witnessing consciousness (puruṣa) distinct from the witnessed (prakṛti). The citta (mind-stuff) comprises three guṇas: sattva (luminosity and clarity), rajas (activity and passion), and tamas (inertia and darkness). Liberation occurs when puruṣa recognizes its eternal distinction from prakṛti’s transformations, including the most subtle mental states. The eight limbs (aṣṭāṅga) systematically reduce identification with body (through yama, niyama, and āsana), breath (prāṇāyāma), senses (pratyāhāra), and mind (dhāraṇā, dhyāna, and samādhi). According to Liberation Mathematics, this tradition correctly identifies identification-with-fluctuation as the mechanism of bondage. However, the endpoint of kaivalya (isolation) diverges from Liberation Mathematics’ emphasis on engagement: whereas Yoga seeks separation from prakṛti, Liberation Mathematics requires continuous domain expansion through worldly participation.

#### 3.2.2 Operational problems

The operational problems include the following questions:

- How can embodied consciousness separate from the material substrate?
- Is complete detachment compatible with social functioning?
- How to measure the ‘separation’ degree objectively?

### 3.3 Buddhism (cessationism)

Traditional formulation: Liberation (nirvana) represents cessation of suffering through elimination of craving (Bodhi, 2000; Rahula, 1974).

#### 3.3.1 Mathematical expression

The mathematical expression defines liberation in Buddhism as,

$$\begin{array}{l} L\_\text{Buddhism}=\text{Suffering}(t)\to 0\;\text{when}:\\ \left[\text{Craving}(t)\to 0]\wedge [\text{Ignorance}(t)\to 0\right] \end{array}$$

#### 3.3.2 Key claims

Buddhism advances four central claims: no permanent self exists (anatta);liberation = ‘blowing out’ of craving; liberation is attainable through systematic practice; and liberation is compatible with embodied existence.

Theoretical foundation: The Buddha’s dependent origination (pratītyasamutpāda) reveals that suffering arises through a twelve-link chain beginning with ignorance (avidyā) and culminating in birth, aging, and death (jarāmaraṇa). Each link conditions the next: ignorance conditions formations, formations condition consciousness, and so forth. The three marks of existence—impermanence (anicca), suffering (dukkha), and non-self (anattā)—systematically undermine identity-fixation by demonstrating that nothing provides a stable ground for identification. The ‘blowing out’ metaphor (nibbāna) suggests not annihilation but the extinguishing of the fires of greed, hatred, and delusion that fuel the cycle. According to Liberation Mathematics, Buddhism’s no-self doctrine aligns closely with PF → 0, and its practical path (magga) offers concrete methods. The apparent paradox—'who achieves liberation if no self exists?’—is resolved by Liberation Mathematics: liberation is not achieved by an agent but constitutes the ongoing dissolution of agent-fixation itself. What remains is nothing but an unfixated awareness continuous with experience.

#### 3.3.3 Operational problems

The operational problems include the following questions:

- If no self exists, who achieves liberation?
- Is complete elimination of desire possible or adaptive?
- How to verify cessation of suffering?

### 3.4 Dualist traditions (Dvaita Vedanta and Jainism)

Dvaita vedanta: Liberation represents eternal loving union with the personal God while maintaining distinction (Sharma, 2000).

L_Dvaita=lim(bhakti→∞)[Union withGod]∧[Self≠God]

Jainism: Liberation through complete karmic purification, achieving omniscient knowledge (Jaini, 1979; Tatia, 1994).

L_Jain=Soul_Purity→100%when Karma→0

Theoretical Foundation—Dvaita: Madhva’s pañca-bheda (five-fold difference) maintains eternal ontological distinction between: (1) God and individual souls, (2) God and matter, (3) individual souls and matter, (4) one soul and another, and (5) one material entity and another. Liberation (mukti) does not dissolve these distinctions but transforms the soul’s relationship to God through bhakti (devotion). The soul, eternally dependent on Lord Vishnu, achieves liberation not through identity-merger but through loving service in eternal proximity to the divine. Gradations of liberation (tāratamya) acknowledge that souls retain individual characteristics even in moksha. According to Liberation Mathematics, Dvaita correctly preserves functional individuality (EC retained) while transforming relational positioning—the soul’s ‘position’ vis-à-vis God shifts from ignorance to devotion without identity dissolution.

Theoretical Foundation—Jainism: Jaina philosophy treats karma as a subtle material substance (karma-pudgala) that literally adheres to and weighs down the soul (jīva). Eight types of karma obstruct the soul’s inherent qualities: knowledge-obscuring, perception-obscuring, feeling-producing, deluding, lifespan-determining, body-determining, status-determining, and obstructive karma. Liberation (moksha, kevala) occurs when all karmic matter is exhausted through right faith (samyak-darśana), right knowledge (samyak-jñāna), and right conduct (samyak-cāritra)—the Three Jewels. The liberated soul (siddha) rises to the apex of the universe, possessing infinite knowledge, infinite perception, infinite bliss, and infinite energy. According to Liberation Mathematics, Jainism’s emphasis on karmic purification corresponds to the removal of conditioning factors that produce PF; however, its endpoint of static perfection diverges from perpetual process.

#### 3.4.1 Operational problems

The operational problems include the following questions:

- How to reconcile eternal distinction with complete union?
- How to verify divine grace or karmic purification objectively?
- What constitutes ‘omniscient knowledge’ operationally?

### 3.5 Western phenomenology (existential freedom)

Phenomenological tradition (Husserl, 1931; Heidegger, 1962; Merleau-Ponty, 1962) addresses authentic existence and transcendence of objectifying consciousness.

#### 3.5.1 Key concepts

The four main key concepts include: reduction (epoché) suspending natural attitude; authentic existence versus das Man (the They); freedom as perpetual transcendence of facticity; and body–subject as a pre-reflective ground.

Theoretical foundation: Husserl’s epoché—the methodological suspension of our everyday assumption that objects exist independently of consciousness—parallels Liberation Mathematics’ recognition that identity-positions are constituted rather than discovered. By bracketing the ‘natural attitude’ toward selfhood, phenomenological reduction reveals that what we take as fixed identity is continually constituted through intentional acts of consciousness. This corresponds directly to the Liberation Mathematics insight that position-fixation (PF) represents not the natural state of consciousness but an acquired rigidity amenable to dissolution.

Heidegger’s analysis of das Man (the ‘They’) illuminates how Dasein (human existence) typically loses itself in anonymous social conformity—'one does this,’ ‘one believes that,’ and ‘one does not question such things.’ This mode of existence, characterized by idle talk (Gerede), curiosity (Neugier), and ambiguity (Zweideutigkeit), represents what Liberation Mathematics formalizes as PF → maximum: identity solidified around inherited cultural positions rather than authentic self-determination. Authentic existence (Eigentlichkeit) emerges through anxiety (Angst), which individualizes Dasein by confronting it with its most fundamental possibilities, including death. According to Liberation Mathematics, Heidegger’s authentic/inauthentic distinction maps onto liberated/imprisoned: authenticity requires perpetual retrieval (Wiederholung) of one’s existence from the They-self, never achieving final possession.

Sartre’s formulation that consciousness perpetually surpasses its given situation—'existence precedes essence’—provides philosophical grounding for Condition 1 (*dE*/*dt* > 0). The for-itself (pour-soi) is defined by what it is not yet, by its projection toward possibilities. Any attempt to coincide with oneself, to become a fixed thing (en-soi-pour-soi), represents bad faith (mauvaise foi). Liberation, in Sartrean terms, is not a state to be achieved but the ongoing refusal to crystallize into thinghood.

**Figure 4:**
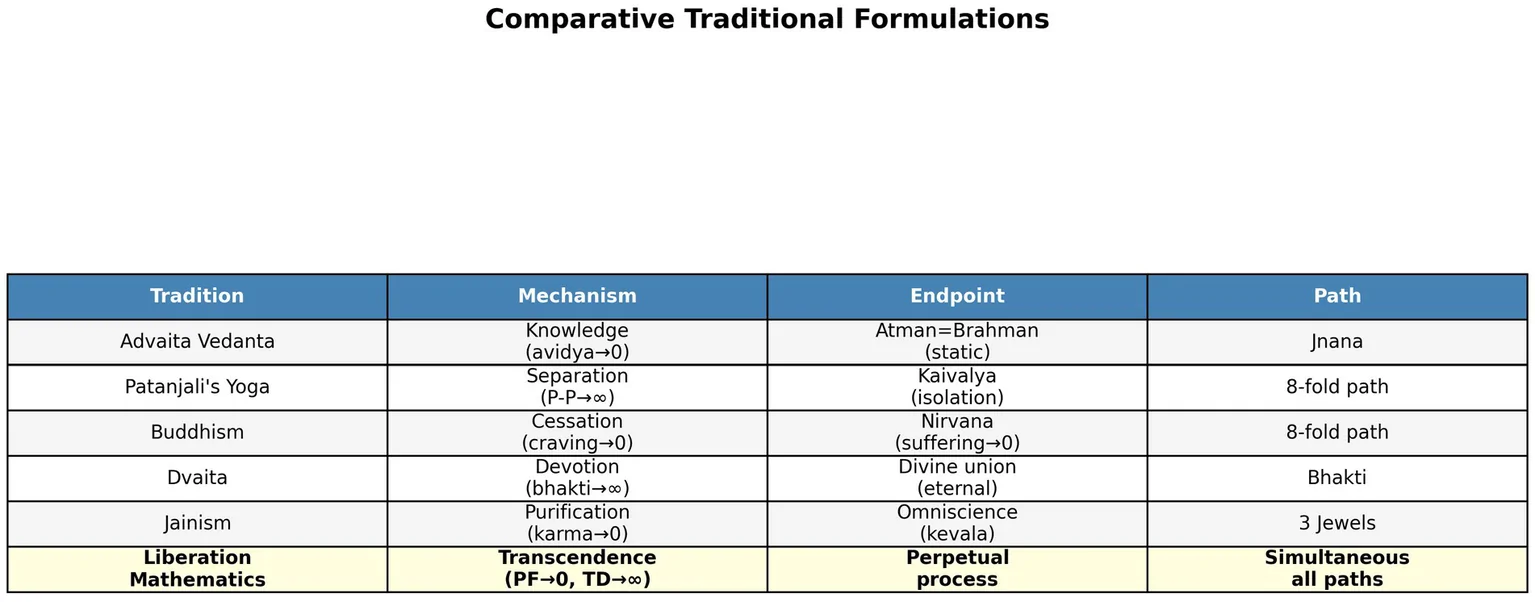
Comparative table contrasting traditional formulations (Advaita, Yoga, Buddhism, Dvaita, and Jainism) versus Liberation Mathematics across key dimensions.

Merleau-Ponty’s body–subject demonstrates that consciousness is always already embodied, neither pure spirit nor mere mechanism. The lived body (corps vécu) is the pre-reflective ground from which all experience arises, challenging both the idealist dissolution of body into consciousness and the materialist reduction of consciousness to body. According to Liberation Mathematics, Merleau-Ponty’s account of the body-subject supports the position that ego capability (EC) remains retained even as PF approaches zero—liberation transforms the ego’s relationship to identity without eliminating functional embodiment. The body–subject continues to navigate the world with acquired skills intact while the reflective self-concept maintains fluidity (Figure 4).

#### 3.5.2 Operational problems

The operational problems include the following questions:

- Can authenticity be sustained or only momentarily achieved?
- How to operationalize ‘authentic’ versus ‘inauthentic’?

#### 3.5.3 Section 3 summary: why traditional models require reformulation?

*Each tradition examined captures genuine dimensions of liberation: Advaita highlights that ignorance constructs a false identity through superimposition; Yoga recognizes that consciousness can witness without identification; Buddhism teaches that craving perpetuates suffering through dependent origination; Dvaita emphasizes relational transformation while preserving individual functionality; Jainism focuses on karmic conditioning as the material substrate of bondage; and Phenomenology analyzes authentic existence as perpetual transcendence of facticity. However, none provides a complete operational model amenable to empirical investigation. Each either lacks empirical tractability (how to verify the atman–Brahman identity?), assumes problematic metaphysical commitments (substantial dualism and material karma), or fails to address all five controversies simultaneously. Liberation Mathematics synthesizes these partial insights into a unified framework by identifying their common operational core—the transcendence of PF—while providing measurable criteria absent from traditional formulations. The following section demonstrates how this synthesis resolves the five traditional controversies.*

## 4 Resolution of traditional controversies

### 4.1 Controversy 1: state versus process

Resolution: Liberation constitutes a dynamic process incapable of termination, not a static achievement.

#### 4.1.1 Mathematical proof

The mathematical proof includes,

- Liberation, when defined as L = Terminal_State, then *dE*/*dt* = 0 (evolution ceases).
- But liberation, L requires *dE*/*dt* > 0 (by definition), which remains a contradiction.
- Therefore: L = Perpetual_Process.

The logical structure of this resolution: (1) Individuals assume that liberation is a terminal state—a condition that, once achieved, persists without further development. (2) A terminal state implies zero evolution rate: if you have ‘arrived,’ movement ceases (*dE*/*dt* = 0). (3) However, the definition of liberation requires a positive evolution rate (*dE*/*dt* > 0)—liberation is essentially the ongoing process of self-transcendence. (4) Therefore, by contradiction, liberation cannot be a terminal state. (5) Conclusion: Liberation must be a perpetual process. The very concept of ‘having achieved liberation’ is incoherent within this framework; individuals who claim final attainment have, by that claim, demonstrated imprisonment in the identity of ‘one who has attained.’

Supporting evidence:

The supporting evidence includes the following:

1. Claims of ‘final enlightenment’ empirically demonstrate subsequent stagnation (LC → 0).
2. Historical ‘enlightened masters’ showing developmental arrest post-declaration.
3. Neurophysiological evidence: brain plasticity continues regardless of attainment (Draganski et al., 2004; Doidge, 2007).

Implication: Traditionally, ‘final states’ actually describe stages appearing final from lower developmental positions, or imprisonment in spiritual identity.

### 4.2 Controversy 2: ego dissolution versus transformation

Resolution: Liberation involves neither ego dissolution nor transformation, but the inability to fix ego positioning.

#### 4.2.1 Liberated ego state

The liberated ego state is attained when the following parameters are met.

- Ego capability: Present (biological/social functioning maintained).
- Ego position: Non-fixable (continuously relocating).
- Ego identification: Impossible (no solidification around role).

#### 4.2.2 Mathematical characterization

The mathematical characterization of PF is given by,

PF(t)→0whileEC(t)=Retained

#### 4.2.3 Observable example

Surgeon experiencing patient vulnerability during surgery while maintaining technical precision—simultaneous multi-perspectival positioning without fixed ‘superior doctor’ identity. The surgeon’s hands perform with EC (retained) while awareness includes the patient’s fear, the family’s hope, and recognition of the surgery’s cosmic insignificance—all without losing the thread of surgical action. This is ego fluidity, not dissolution.

### 4.3 Controversy 3: world negation versus affirmation

Resolution: Liberation mandates maximum world engagement, not withdrawal.

Mathematical proof:

The mathematical proof is given by,

Liberation function:L(t)∝∫TD(t)dt

where TD(*t*) is the testing domains encountered. Further,

when Engagement(*t*) → 0 (withdrawal):

then TD(*t*) → Limited_Set.

But L requires: TD(t) → ∞ remains a contradiction.

Therefore, L requires Engagement(*t*) → Maximum.

Rationale

The rationale includes:

1. Each domain represents a unique testing ground for PF detection.
2. Withdrawal limits domain variety, enabling undetected fixations.
3. Maximum engagement provides maximum feedback.
4. Historical evidence: cave-dwelling individuals often demonstrate imprisonment in ‘renunciant’ identity.

Clarifying example: The cave-dwelling renunciant who identifies as ‘one who has transcended worldly attachment’ remains imprisoned—now within spiritual rather than material identity. The renunciant’s PF has not approached zero but merely shifted object, from ‘successful businessman’ to ‘transcendent sage.’ Maximum world engagement provides maximum testing domains, exposing latent PF that isolation would leave undetected. The businessman navigating ethical complexity, the parent surrendering professional identity for childcare, and the expert entering domains of incompetence—each encounters tests unavailable to those who withdraw.

### 4.4 Controversy 4: path primacy

Resolution: All paths represent necessary simultaneous requirements, not alternatives.

$$L(t)=\int [\begin{array}{l} \text{Knowledge}(t)\times \text{Devotion}(t)\\ \times \text{Action}(t)\times \text{Meditation}(t) \end{array}]\;\mathrm{dt}$$

Redefinitions in liberation mathematics:

The various redefinitions in liberation mathematics include:

- Knowledge (Jnana): Fresh perception with retained capability.
- Devotion (Bhakti): Complete surrender to perpetual self-transcendence.
- Action (Karma): Behaviors actively negating own position.
- Meditation (Dhyana): Fresh perception maintenance amid expert action.

Implication: Sectarian conflicts claiming one path’s superiority reflect incomplete models. The multiplication sign (×) in the formula indicates that when any path becomes zero, the entire product equals zero—liberation requires all paths simultaneously.

### 4.5 Controversy 5: sudden versus gradual

Resolution: Liberation involves three temporally distinct components, namely:

$\begin{array}{l} \text{Liberation}=\text{Recognition}(\text{sudden})⊗\text{Application}(\text{continuous})\\ ⊗\text{Capability}(\text{gradual}) \end{array}$

Observable pattern:

The stages of observable patterns include:

1. Stage 1 (Gradual): Years of expertise accumulation.
2. Stage 2 (Sudden): Recognition: ‘My expertise imprisons me’.
3. Stage 3 (Continuous): Ongoing fresh perception application.
4. Stage 4 (Perpetual): No terminus—continuous self-transcendence.

**Figure 5:**
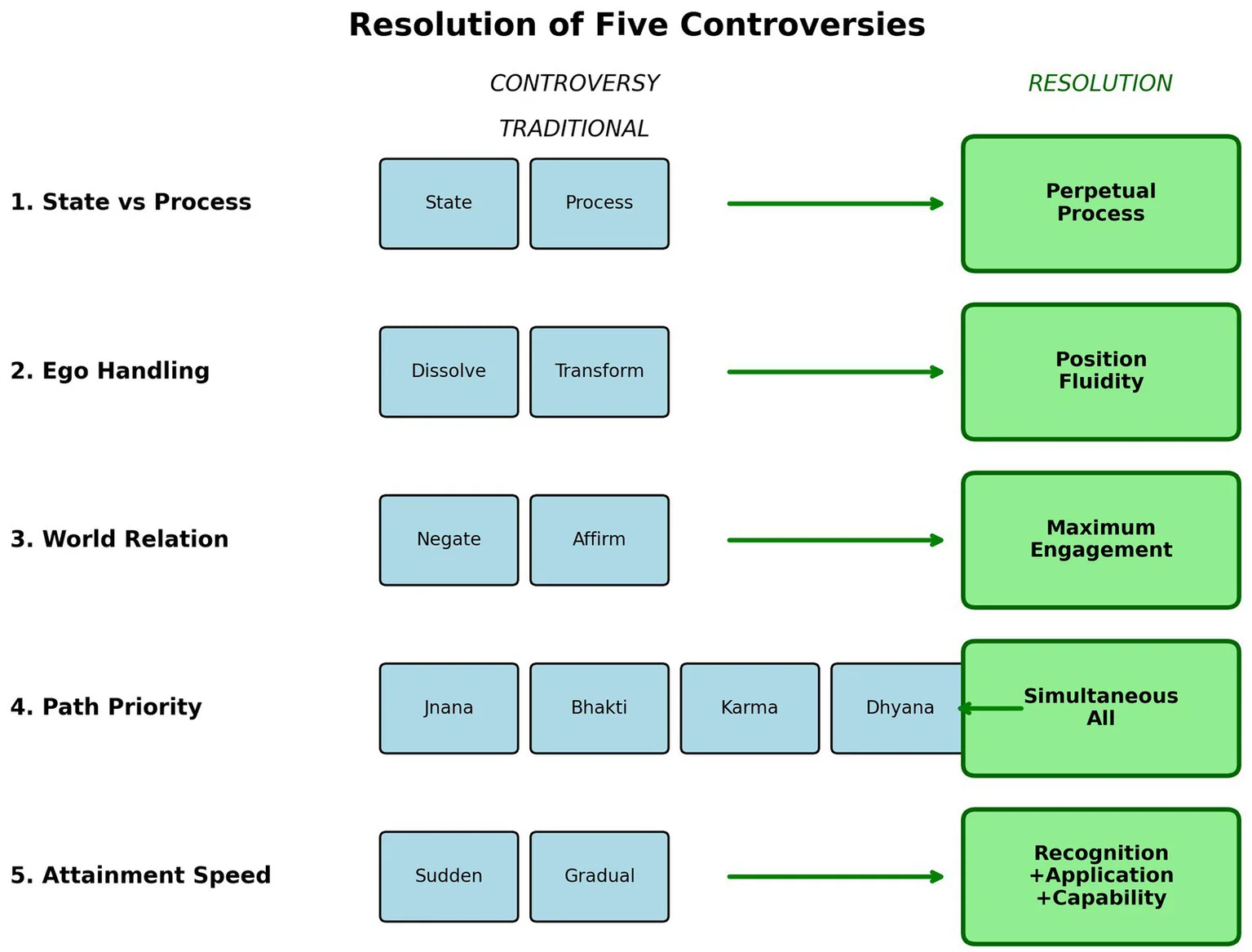
Visual summary of how Liberation Mathematics resolves all five traditional controversies through a unified framework.

Implication: Debate represents a false dichotomy. Both are necessary components. The Zen tradition’s emphasis on sudden awakening (satori) captures Stage 2; the Theravada emphasis on gradual path (magga) captures Stages 1 and 3–4. Liberation Mathematics integrates both by distinguishing recognition, application, and capability as separate temporal dimensions of a unified process (Figure 5).

#### 4.5.1 Section 4 summary: unification through process

*The five traditional controversies dissolve once liberation is reconceived as a perpetual process rather than a terminal state. State versus process: liberation cannot terminate without becoming imprisonment, because terminus implies cessation of evolution (dE/dt = 0), which contradicts the definition. Ego dissolution versus transformation: the ego remains functionally present (EC retained) but positionally unfixed (PF → 0)—neither destroyed nor merely transformed but rendered fluid. World negation versus affirmation: maximum engagement provides maximum testing domains (TD → ∞), while withdrawal enables undetected fixations. Path primacy: the multiplication formula requires all paths simultaneously—if any equals zero, liberation equals zero. Sudden versus gradual: recognition is sudden, application continuous, capability gradual—the apparent contradiction reflects conflation of distinct temporal components. These apparent contradictions, which have generated millennia of sectarian dispute, reflected incomplete perspectives on a unified phenomenon whose mathematical formalization reveals their complementarity. The following section develops empirical protocols for testing this framework's predictions.*

## 5 Empirical validation framework

### 5.1 Behavioral assessment protocol

Objective: The behavioral assessment quantifies liberation through observable behavioral patterns.

Method: Six-domain behavioral testing across a 30-day period.

Test battery:

*Test 1: Achievement response*. Present a significant personal achievement. Measure: Time before pursuing greater challenge. Scoring: Liberated (T < 24 h), Intermediate (24 h < T < 7 days), and Imprisoned (T > 7 days).

*Test 2: Authority negation*. Place the subject in an authority position. Measure: Actions toward eliminating that position. Scoring: Liberated (active system-building to replace self), Intermediate (ambivalent position maintenance), and Imprisoned (authority consolidation).

*Test 3: Recognition response*. Provide formal recognition/award. Measure: Reference frequency in communications. Scoring: Liberated (zero references), Intermediate (occasional references), and Imprisoned (frequent references).

*Test 4: Expert demonstration*. Request an explanation of domain expertise. Measure: Book/credential versus natural phenomena references. Scoring: Liberated (demonstrates via nature), Intermediate (mixed approach), and Imprisoned (credential emphasis).

*Test 5: Failure integration*. Present domain-specific failure/refutation. Measure: Defensive response versus integration. Scoring: Liberated (welcomes correction), Intermediate (reluctant acceptance), and Imprisoned (defensive).

*Test 6: Hierarchy maintenance*. Interact with perceived ‘inferiors.’ Measure: Superior position versus authentic receptivity. Scoring: Liberated (position fluidity), Intermediate (polite but hierarchical), and Imprisoned (fixed superiority).

Composite liberation coefficient:

The composite LC is given by,

LC_behavioral=(T1+T2+T3+T4+T5+T6)/6

The interpretations of LC indicate that when LC ≥ 0.8 (high liberation), 0.5 ≤ LC < 0.8 (moderate liberation), and LC < 0.5 (low liberation/imprisonment).

### 5.2 Neurophysiological markers

Objective: Identify neural correlates of liberation state.

Method: Functional magnetic resonance imaging (MRI) during liberation-relevant tasks.

Predicted neural signatures:

The imprisoned state markers include:

1. Elevated Default Mode Network (DMN) activity (Gusnard et al., 2001).
2. Increased medial prefrontal cortex (mPFC) activation to achievement stimuli (Northoff et al., 2006).
3. Reduced cognitive flexibility markers (anterior cingulate cortex) (Leber et al., 2008).
4. Heightened defensive responses (amygdala activation) (Izuma et al., 2010).

#### 5.2.1 Liberation state markers

**Figure 6:**
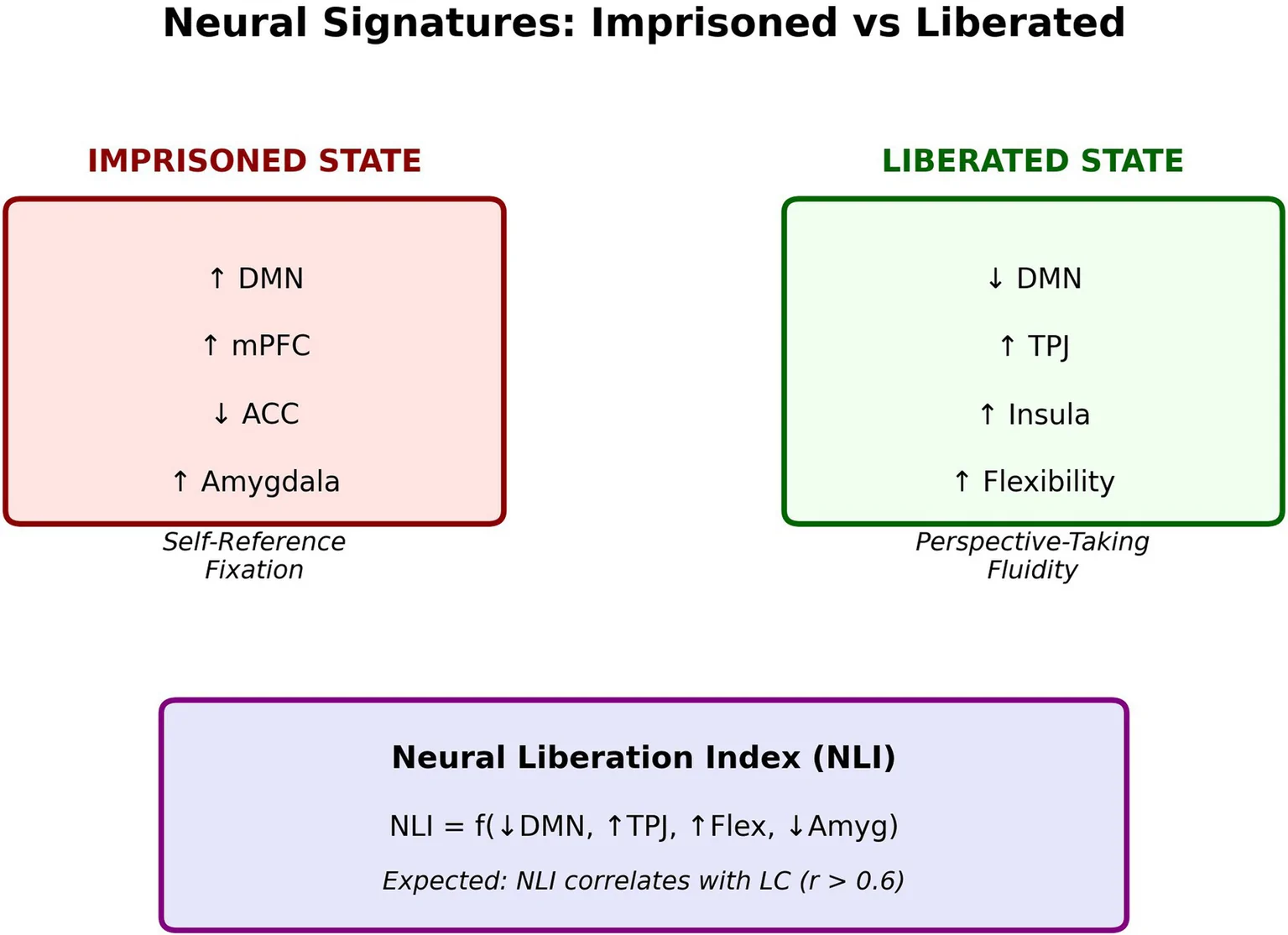
Predicted neurophysiological differences between imprisoned and liberated consciousness states during self-reference tasks.

The five liberation state markers include:

1. Reduced DMN activity during self-reference.
2. Increased temporoparietal junction (TPJ) activation (perspective-taking) (Saxe and Kanwisher, 2003).
3. Enhanced insular cortex activity (interoceptive awareness) (Craig, 2009).
4. Increased network flexibility indices (Bassett et al., 2011).
5. Reduced amygdala reactivity to identity challenges (Figure 6).

#### 5.2.2 Experimental protocol

The experimental protocols include:

Session 1: Baseline neural measures (30-min scanning session)—Resting state connectivity (8 min), self-reference task activation (10 min), and achievement-related stimuli response (12 min).

Session 2: Liberation training (30 days)—Daily practice of PF exercises, domain expansion activities, and achievement-transcendence protocols.

Session 3: Post-training neural measures (30-min scanning session)—Same tasks as baseline, compare pre/post patterns.

Statistical validation:

- The minimal sample size includes a pilot study *n* ≈ 30 (feasibility assessment) and a complete validation *n* ≈ 100 (power = 0.8 at *α* = 0.05).
- The analysis involves calculating the Neural Liberation Index (NLI).
- The expected outcome showed that NLI increases proportionally to LC (r > 0.6), and the timeline shows a 6-month data collection per cohort.

### 5.3 Longitudinal development protocol

Objective:

The main objective involves mapping liberation development trajectory over extended timeframes.

Method:

The method comprises a 6-month intensive monitoring with monthly assessments.

Measurement schedule:

Baseline (Month 0): Complete behavioral battery, neurophysiological assessment, self-report questionnaires, and domain inventory (current expertise areas).

Monthly Assessments (Months 1–6): LC calculation, STR measurement, new domain entry count, achievement transcendence latency, and PF incidents.

Predicted developmental trajectory:

The mathematical model of LC development shows that

dLC/dt=λ·LC·(1−LC)

This logistic growth equation predicts slow initial progress (low LC), rapid middle phase (0.3 < LC < 0.7), and asymptotic approach when LC = 1.

**Figure 7:**
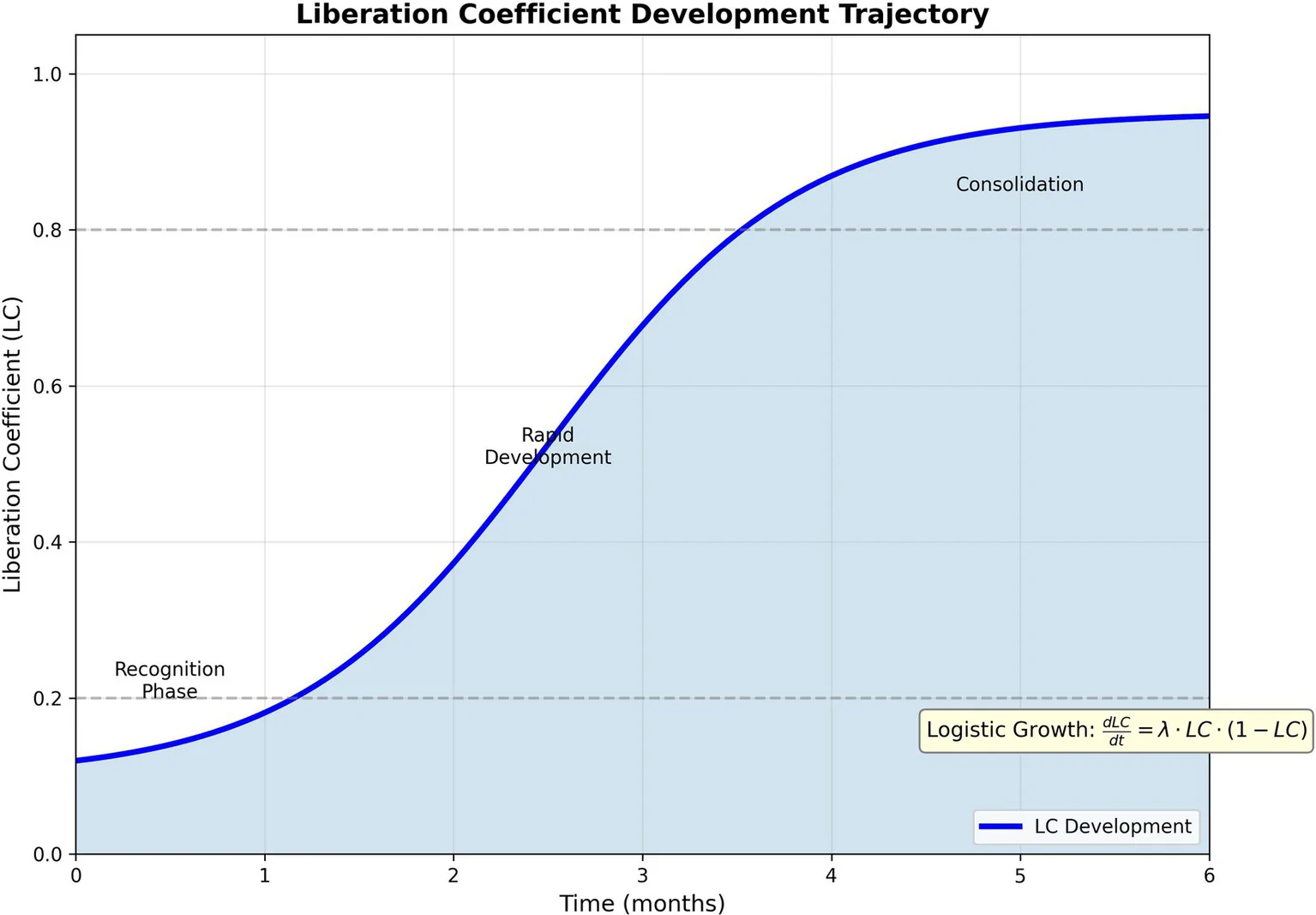
Predicted liberation coefficient development trajectory following logistic growth curve over a 6-month period.

Typical timeline: Month 0–1: LC ≈ 0.2 (recognition phase); Month 2–4: LC ≈ 0.5–0.7 (rapid development); and Month 5–6: LC ≈ 0.8 + (consolidation) (Figure 7).

#### 5.3.1 Section 5 summary: from theory to testability

*This section has demonstrated that Liberation Mathematics generates falsifiable predictions testable with current methods. The behavioral assessment protocol yields LC scores from observable actions (achievement response latency, authority negation behavior, and recognition reference frequency), not subjective self-report. The neuroimaging protocols predict specific neural signatures distinguishing imprisoned from liberated states (elevated DMN vs. reduced DMN, mPFC self-reference activation vs. TPJ perspective-taking), testable with standard fMRI paradigms. The longitudinal protocol predicts logistic growth curves for LC development, generating specific quantitative predictions (LC ≈ 0.2 at month 1, LC ≈ 0.7 at month 4). These predictions are falsifiable: if trained individuals show no increase in LC, when neural patterns fail to correlate with behavioral measures or when developmental trajectories deviate substantially from logistic curves, the framework requires revision. The LC, STR, and NLI translate abstract formalization into quantifiable data, enabling systematic empirical investigation of consciousness liberation.*

## 6 Practical implementation

Having established the theoretical framework and its empirical testability, this section outlines practical protocols for liberation cultivation across individual and institutional contexts.

### 6.1 Daily liberation practice protocol (summary)

Objective: Provide a systematic method for liberation cultivation accessible to general population.

**Figure 8:**
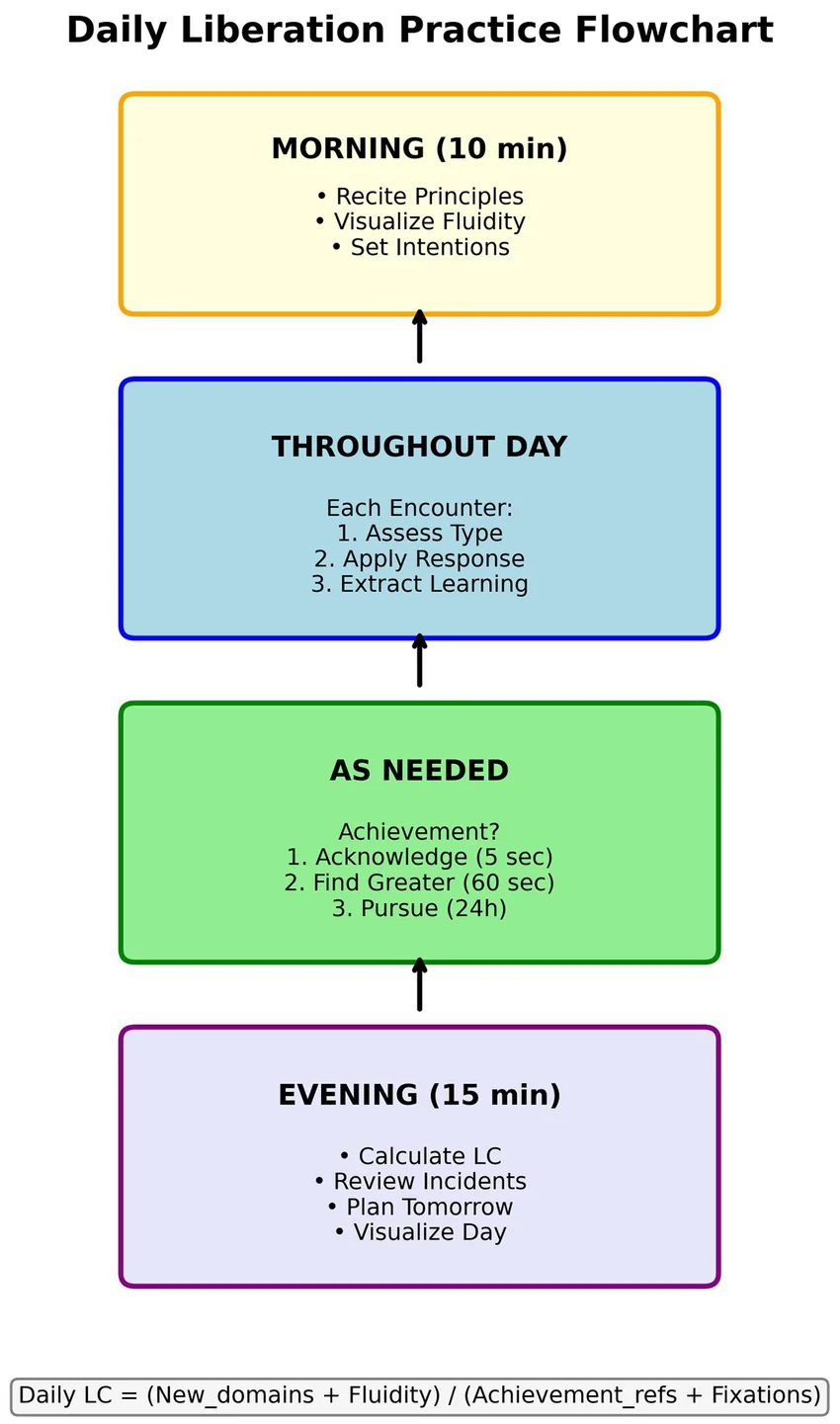
Daily liberation practice protocol flowchart showing four-component structure for systematic cultivation.

Structure: The four-component daily practice (see Figure 8 for complete flowchart) includes,

*Component 1: Morning Recognition (10 min)* includes recitation of liberation principles, identity fluidity visualization, and intention setting for perpetual transcendence.

*Component 2: Throughout-day application* includes encounter-type assessment (inferior/superior/equal), position-fluidity maintenance, and real-time learning extraction.

*Component 3: Achievement Transcendence (as needed)* includes immediate acknowledgment (5 s), greater challenge identification (60 s), and pursuit initiation (within 24 h).

*Component 4: Evening integration (15 min)* includes daily LC calculation, critical incident review, planning for tomorrow, and day visualization as observer.

### 6.2 Education implementation

Objective: The main objective of education implementation is to integrate liberation mathematics into educational curricula across developmental stages.

Proposed curriculum modifications:

Primary education (Ages 6–12): Expert-beginner mind through mathematics, position fluidity through role reversal games, and celebrating being proven wrong as growth.

Secondary education (Ages 13–18): Explicit teaching of liberation principles, regular domain expansion assignments, and achievement transcendence as core competency.

Higher education (Ages 18+): Liberation Mathematics as a formal discipline, research training in Liberology methods, and integration with contemplative studies programs.

Professional training: Medical: Surgeon–patient position fluidity; Legal: Judge reducing judicial necessity; Education: Teacher creating superior students; and Business: Leader building self-replacing systems.

## 7 Discussion

### 7.1 Philosophical implications

#### 7.1.1 Mind–body problem resolution

The proposed model addresses mind–body dualism (Chalmers, 1996; Kim, 2005) by recognizing consciousness as simultaneously embodied (requiring ego for biological function) and trans-personal (incapable of PF). This transcends both eliminativist materialism and substance dualism, aligning instead with embodied cognition frameworks (Thompson, 2015).

#### 7.1.2 Free will paradox

Liberation mathematics may provide a resolution to free will debates (Wegner, 2002; Kane, 2005) by demonstrating freedom as non-fixation of positioning rather than uncaused causation. The liberated individual acts freely through the inability to imprison action within fixed identity structures, consistent with compatibilist frameworks.

#### 7.1.3 Infinite regress problem

Traditional enlightenment discourse suffers from infinite regress: ‘Who achieves enlightenment if no self exists?’ Liberation mathematics resolves this by eliminating achievement entirely—liberation is an ongoing process preventing fixation, not a state attained by an agent.

#### 7.1.4 Authenticity problem

Phenomenological concern with authentic existence (Husserl, 1931; Heidegger, 1962; Merleau-Ponty, 1962) finds operational definition: authenticity = position-fluidity (liberation) and inauthenticity = PF (imprisonment). This moves authenticity from existential anxiety to a measurable behavioral phenomenon.

### 7.2 Relationship to contemporary science

#### 7.2.1 Predictive processing framework

Recent predictive processing models (Clark, 2013; Friston, 2010, 2019) align with liberation mathematics. PF represents over-reliance on prior beliefs; liberation represents perpetual Bayesian updating without consolidation of believes. This connects to Active Inference frameworks (Friston, 2019) where liberation corresponds to minimizing free energy while maximizing epistemic value exploration.

#### 7.2.2 Integrated information theory

Liberation may relate to maximal integrated information (*Φ*) with minimal rigidity (Tononi, 2016; Seth, 2021). The liberated state maintains high integration (coherent experience) while enabling rapid reconfiguration (perspective fluidity), representing an optimal balance between integration and flexibility.

#### 7.2.3 Neuroplasticity research

Evidence for lifelong neuroplasticity (Draganski et al., 2004; Doidge, 2007) supports the process model against terminal state formulations. The brain continues reorganizing regardless of contemplative attainment, consistent with the perpetual transcendence requirement.

#### 7.2.4 Developmental Psychology

Post-conventional stage models (Cook-Greuter, 2000; Kegan, 1994) parallel liberation mathematics’ emphasis on self-transcendence, though the models typically frame advanced stages as final rather than continuously evolving. Liberation mathematics extends these models by removing the developmental ceiling.

#### 7.2.5 Cognitive flexibility

Research on cognitive flexibility and switching costs (Monsell, 2003; Diamond, 2013) provides a mechanistic understanding of PF. Liberation represents maximized flexibility—minimized switching costs between perspectives. This connects to Barrett et al.’s (2024) work on metacognitive flexibility as a predictor of adaptive functioning.

### 7.3 Cross-cultural validity

The proposed model demonstrates potential cross-cultural applicability by:

1. Resolving inter-traditional contradictions without privileging any single tradition.
2. Providing neutral operational language translatable across cultural contexts.
3. Identifying behavioral universals rather than culture-specific practices.
4. Enabling empirical validation independent of belief systems.
5. Recognizing all paths as simultaneous requirements rather than cultural alternatives.

Testing across diverse cultural contexts (Western, Hindu, Buddhist, Islamic, and Indigenous) represents a critical next step for validation. The framework’s mathematical formalization may facilitate cross-cultural dialogue by providing shared operational definitions while respecting traditional wisdom.

### 7.4 Integration with contemporary phenomenology

#### 7.4.1 Varela’s enactive cognition

Varela et al.’s (1991) embodied mind framework aligns with liberation mathematics’ emphasis on position-fluidity within embodied existence. Liberation does not transcend embodiment but rather prevents fixation within any particular embodied perspective.

#### 7.4.2 Zahavi’s self-awareness

Zahavi’s (2005) work on pre-reflective self-awareness illuminates how liberated consciousness maintains experiential continuity (non-zero self-awareness) while preventing identity crystallization (zero PF).

### 7.5 Limitations and future directions

#### 7.5.1 Current Limitations include

1. Theoretical Foundation: The model requires empirical validation through proposed experimental protocols.
2. Longitudinal Data: Developmental trajectories need multi-year tracking beyond a 6-month pilot study.
3. Cultural Validation: Cross-cultural testing has not yet been conducted across diverse traditions.
4. Neural Specificity: Predicted neurophysiological markers require validation with adequate power.
5. Individual Variation: The model may not account for all developmental pathways or alternative trajectories.

## 8 Ethics statement

All future empirical studies derived from this theoretical framework will adhere strictly to international research-ethics standards, including the Declaration of Helsinki (2013 revision) and applicable Institutional Review Board (IRB) requirements. When the Liberation Coefficient (LC) or other Liberation Mathematics variables are applied in populations with psychiatric or identity-related vulnerabilities, participation may occur only under licensed clinical supervision with informed consent, psychological risk screening, and the right to withdraw at any time without penalty. The LC will be treated exclusively as a behavioral-theoretical construct, not as a diagnostic or therapeutic tool. Any indication of distress, dissociative rigidity, or psychotic identification (such as LC < 0.2) will prompt immediate clinical evaluation rather than continued testing. All data will be anonymized, securely stored, and analyzed according to institutional and national ethical regulations.

Future research directions include:

1. Large-scale behavioral validation (*n* > 500 across multiple sites).
2. Neuroimaging validation of predicted markers with adequate power analysis.
3. Longitudinal development tracking (5–10 years minimum).
4. Cross-cultural replication across 5 + distinct traditions (Buddhist, Hindu, Christian contemplative, Islamic Sufi, and Indigenous).
5. Development of Liberation Science (Liberology) as a formal academic discipline with dedicated research programs.
6. Integration with existing contemplative science programs at universities.
7. Educational intervention studies across developmental stages (K-12, higher education, and professional).
8. Professional training program development and assessment in clinical, legal, educational, and business contexts.
9. Connection to psychopathology research by investigating whether LC < 0.2 reliably predicts dissociative or identity disorders.
10. Lifespan development by examining liberation trajectories across the entire lifespan from adolescence through late adulthood.

### 8.1 Methodological refinements needed

- Ecological momentary assessment (EMA) protocols for real-time LC tracking.
- Qualitative phenomenological interviews to complement quantitative measures.
- Machine learning approaches to identify nonlinear developmental patterns.
- Cross-validation of behavioral and neural measures.
- Development of standardized assessment instruments with established psychometric properties.

## 9 Conclusion

This study presents a proposed operational definition of liberation amenable to systematic empirical investigation. By formalizing liberation as perpetual self-transcendence characterized by non-fixation of identity positions, the model:

1. Resolves five major philosophical controversies that have persisted across traditions for over 2,500 years.
2. Provides empirically testable criteria through behavioral and neurophysiological markers.
3. Enables cross-cultural dialogue through neutral operational language.
4. Establishes practical implementation protocols accessible to general populations.
5. May provide foundation for new scientific discipline—Liberation Science (Liberology)—for systematic study of consciousness liberation.

The proposed framework represents neither Eastern mysticism nor Western reductionism, but rather a mathematical formalization of observable behavioral and consciousness phenomena. Liberation emerges not as a supernatural attainment but as a natural developmental trajectory potentially available through systematic practice.

Future work must validate predicted behavioral patterns, neural signatures, and developmental trajectories across diverse populations and extended timeframes. The ultimate test will be whether individuals can systematically cultivate measurably increased Liberation Coefficients through application of proposed protocols, and whether these increases correlate with meaningful improvements in psychological flexibility, life satisfaction, and adaptive functioning.

If validated, Liberation Mathematics may provide a unified scientific framework for 2,500 years of contemplative wisdom, transforming mystical philosophy into testable science while preserving spiritual profundity. This represents not a conclusion but a potential inauguration—the beginning of systematic, empirical Liberation Science pending empirical validation.

The framework’s success depends on careful empirical testing, cross-cultural validation, integration with existing consciousness research programs, and willingness to refine or reject elements that fail empirical scrutiny. This study invites researchers across contemplative traditions, neuroscience, psychology, and philosophy to test, critique, and refine these proposals through rigorous investigation.

## Data Availability

The original contributions presented in the study are included in the article/Supplementary material, further inquiries can be directed to the corresponding author.
