# Correction: Liberation mathematics I: the behavioral and consciousness definition of perpetual self-transcendence

**DOI:** 10.3389/fpsyg.2026.1877906

**Published:** 2026-08-26

**Authors:** Swapan Samanta

**Affiliations:** Indoriv Clinical Research Centre, Kolkata, India

**Keywords:** behavioral mathematics, consciousness, empirical spirituality, liberation, moksha, phenomenology, self-transcendence

In the published article, the figure images and their corresponding captions were misaligned for Figures 3, 4, 5, 6, and 7. The figure images appeared in incorrect positions while the captions remained in their correct locations, resulting in mismatches between figure content and descriptions. Additionally, Supplementary Figure S1 was erroneously included as Figure 5 in the main article.

**Specific errors:**

**Figure 3:** The image displayed was the “Daily Liberation Practice Flowchart” (which should appear as Figure 8), while the caption correctly read “Distinguishing pathological fixation (rigid single identity, L → 0) from genuine liberation (fluid multiple perspectives, L = 50–200/day) under diagnostic criteria.” The correct image for Figure 3 should show the pathological fixation versus genuine liberation diagnostic comparison diagram.

**Figure 4:** The image displayed was the “Liberation Coefficient Development Trajectory” graph showing logistic growth curve (which should appear as Figure 7), while the caption correctly read “Comparative table contrasting traditional formulations (Advaita, Yoga, Buddhism, Dvaita, and Jainism) versus Liberation Mathematics across key dimensions.” The correct image for Figure 4 should show the comparative table of traditional formulations.

**Figure 5:** The image displayed was the “Causal Flow Diagram” (which should appear as Supplementary Figure S1, not in the main article), while the caption correctly read “Visual summary of how Liberation Mathematics resolves all five traditional controversies through a unified framework.” The correct image for Figure 5 should show the visual summary of controversy resolutions. The Causal Flow Diagram should be moved to Supplementary Material as Figure S1.

**Figure 6:** The image displayed was the “Pathological Fixation vs Genuine Liberation” diagram (which should appear as Figure 3), while the caption correctly read “Predicted neurophysiological differences between imprisoned and liberated consciousness states during self-reference tasks.” The correct image for Figure 6 should show the predicted neural signatures diagram with brain regions.

**Figure 7:** The image displayed was the “Resolution of Five Controversies” visual summary (which should appear as Figure 5), while the caption correctly read “Predicted liberation coefficient development trajectory following logistic growth curve over a 6-month period.” The correct image for Figure 7 should show the LC development trajectory graph.

**The correct Figure captions are as follows:**

• Figure 3 should display: Pathological Fixation vs Genuine Liberation diagnostic comparison.

• Figure 4 should display: Comparative table of traditional formulations versus Liberation Mathematics.

• Figure 5 should display: Visual summary of controversy resolutions.

• Figure 6 should display: Predicted neurophysiological differences (neural signatures diagram).

• Figure 7 should display: Liberation Coefficient development trajectory (logistic growth curve).

**Figure 3:**
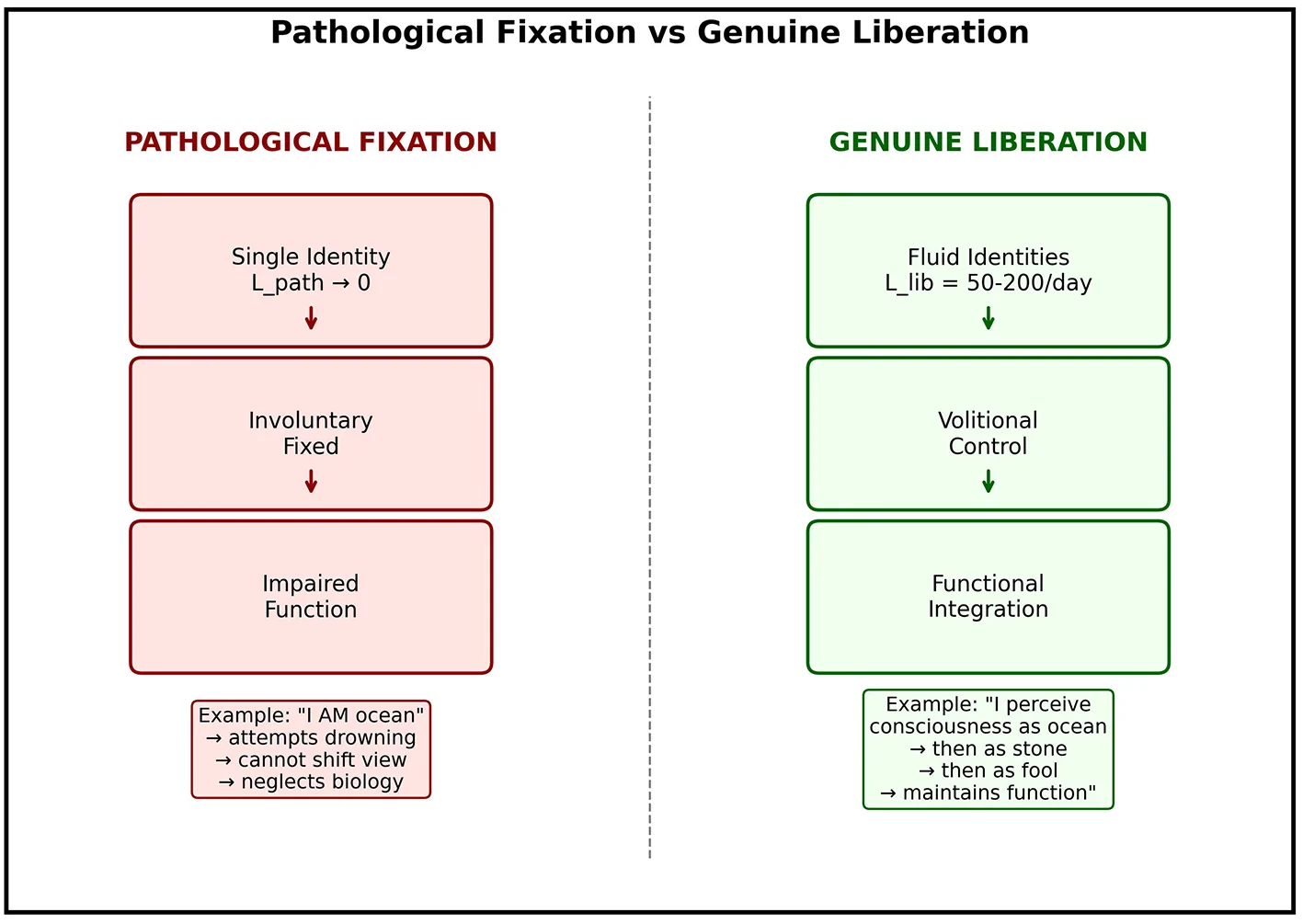
Distinguishing pathological fixation (rigid single identity, L → 0) from genuine liberation (fluid multiple perspectives, L = 50–200/day) under diagnostic criteria.

**Figure 4:**
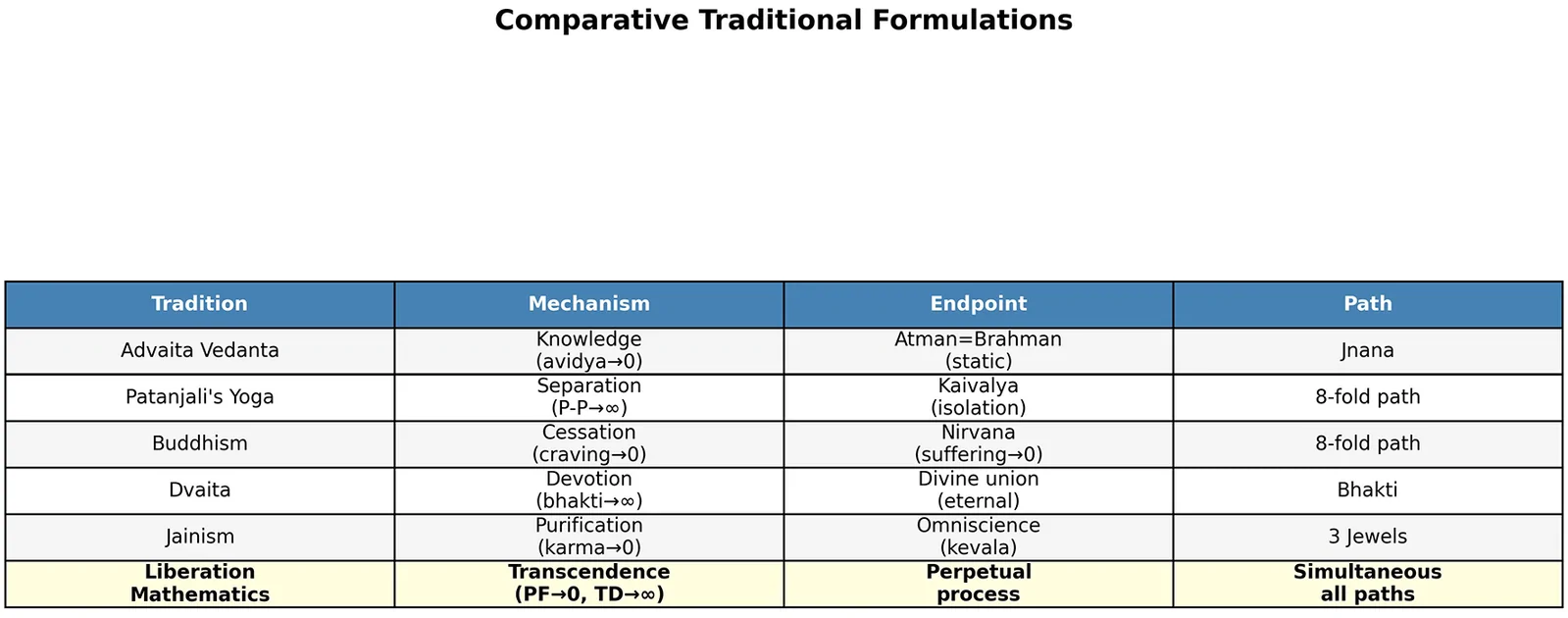
Comparative table contrasting traditional formulations (Advaita, Yoga, Buddhism, Dvaita, and Jainism) versus Liberation Mathematics across key dimensions.

**Figure 5:**
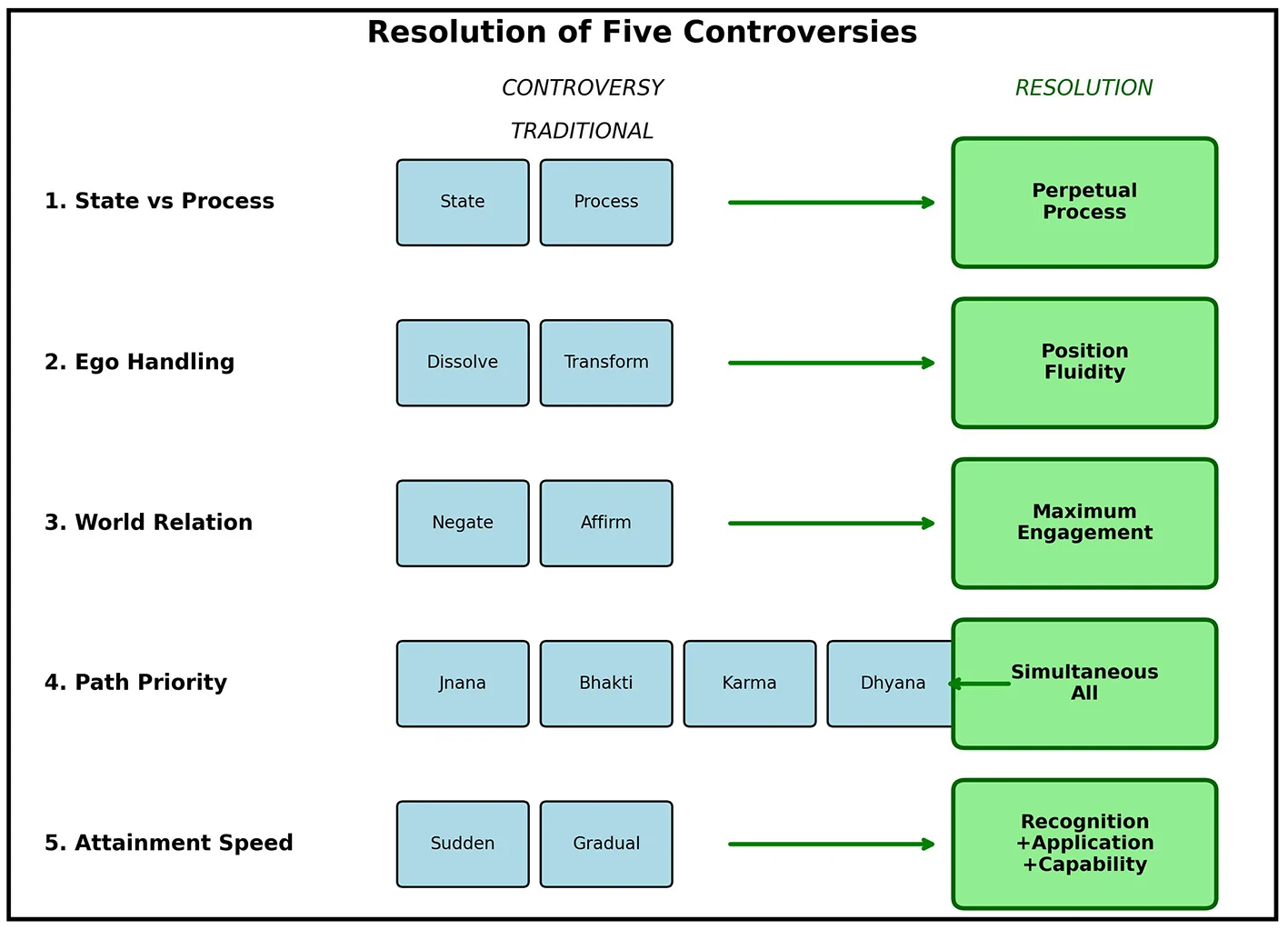
Visual summary of how Liberation Mathematics resolves all five traditional controversies through a unified framework.

**Figure 6:**
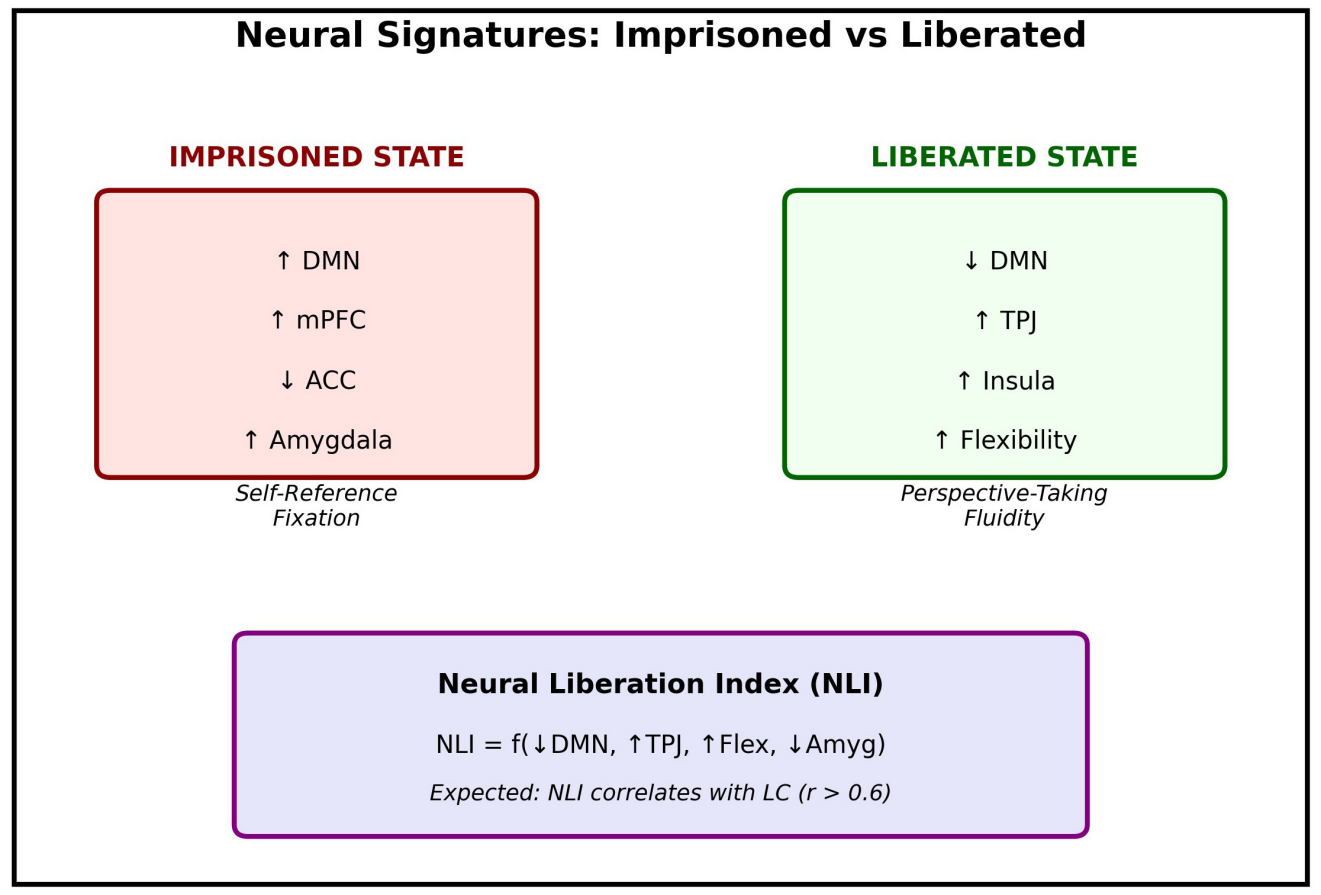
Predicted neurophysiological differences between imprisoned and liberated consciousness states during self-reference tasks.

**Figure 7:**
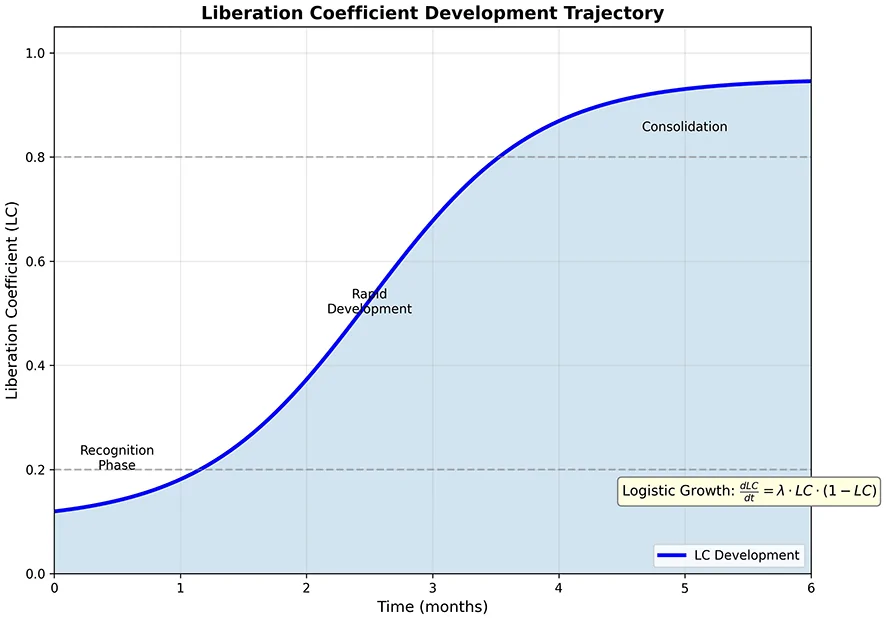
Predicted liberation coefficient development trajectory following logistic growth curve over a 6-month period.

**Figure 8:**
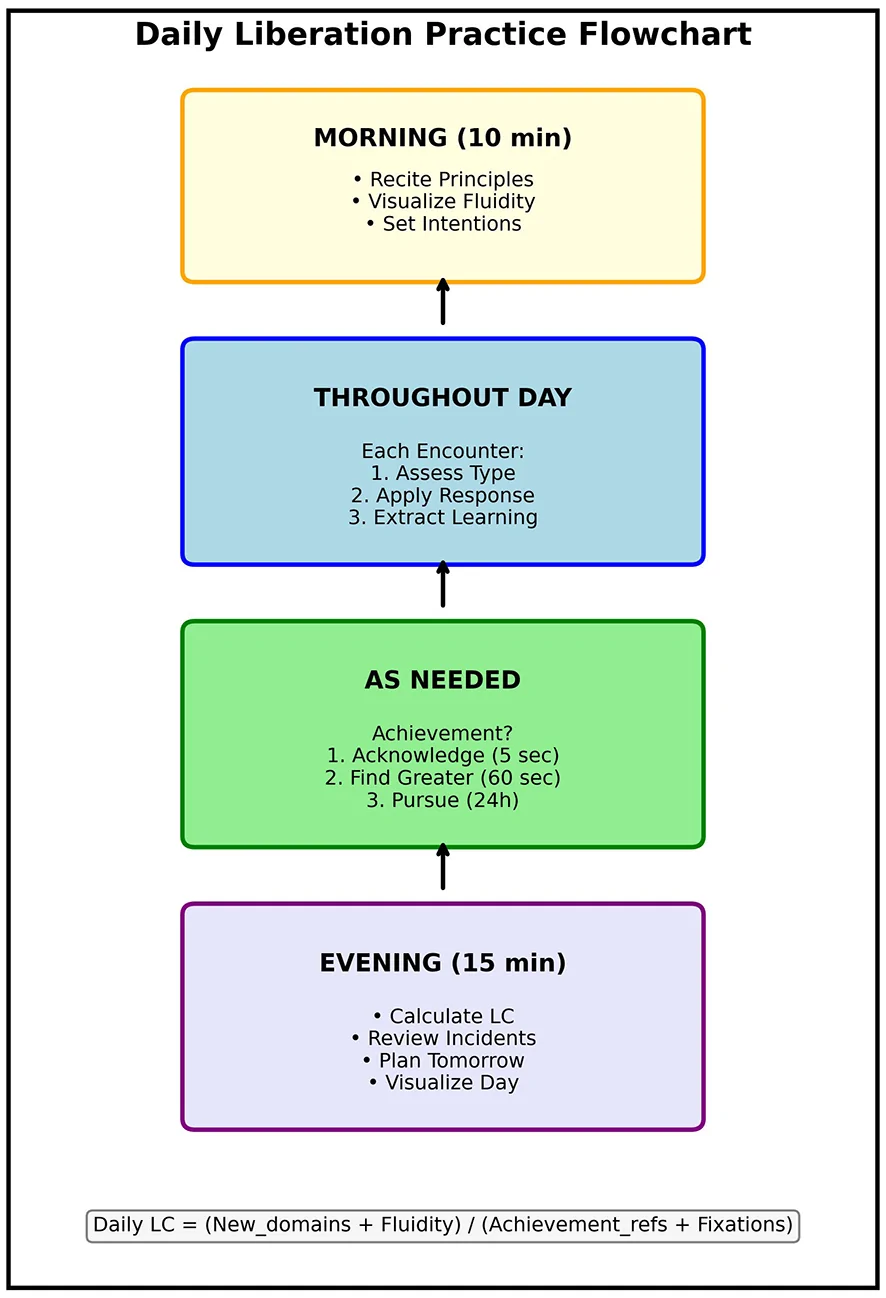
Daily liberation practice protocol flowchart showing four-component structure for systematic cultivation.

• Supplementary Figure S1 should display: Causal Flow Diagram (to be moved from main article to Supplementary Material).

The original version of this article has been updated.

