# Supplementary material for: Liberation mathematics I: the behavioral and consciousness definition of perpetual self-transcendence
Source: Front Psychol. 2026 May 4;17:1741096. doi: 10.3389/fpsyg.2026.1741096 (PMC13180822; doi:10.3389/fpsyg.2026.1741096)
Supplement: Supplementary file 1 [file Supplementary_file_1.DOCX]

**SUPPLEMENTARY MATERIALS**

*Liberation Mathematics I: The Behavioral and Consciousness Definition of Perpetual Self-Transcendence*

Dr. Swapan Samanta, MD

Frontiers in Psychology – Theoretical and Philosophical Psychology

**Contents:**

Supplementary Material S1: Detailed Daily Liberation Practice Protocol

Supplementary Material S2: Liberation Coefficient Calculation Worksheets

Supplementary Material S3: Behavioral Assessment Battery

Supplementary Material S4: Neuroimaging Task Specifications

Supplementary Material S5: Cross-Cultural Validation Protocol

# Supplementary Material S1: Detailed Daily Liberation Practice Protocol

## Introduction and Purpose

This protocol provides a practical, day-by-day guide for cultivating liberation as defined in the Liberation Mathematics framework. Unlike traditional contemplative practices that may emphasize withdrawal or purely internal states, this protocol is designed for active engagement with daily life. The underlying principle is straightforward: liberation is not something we achieve and then possess, but rather a way of moving through the world that prevents identity from crystallizing around any fixed position—whether that position is professional success, spiritual attainment, or anything else.

The protocol requires approximately 25-30 minutes of dedicated practice time daily, distributed across morning and evening sessions, with additional moment-to-moment applications throughout the day. Practitioners should approach this not as another achievement to master, but as an ongoing experiment in living without fixation.

## Component 1: Morning Recognition Practice (10 minutes)

### Setting and Preparation

Find a quiet space where you can sit comfortably for ten minutes without interruption. This need not be an elaborate meditation setup—a kitchen chair, the edge of your bed, or even a parked car will serve. The key is consistency: practicing at the same time and place each morning helps establish the routine without requiring willpower.

### The Recognition Recitation (3 minutes)

Begin by reading aloud or silently reciting the following principles. The purpose is not to memorize doctrine but to orient attention toward the day ahead:

*"Today, I recognize that any position I achieve—any success, any recognition, any state of mind—is not a place to rest but a launching point for further exploration. When I notice myself solidifying around any identity, whether 'expert' or 'beginner,' 'spiritual' or 'worldly,' I will gently release that grip. My goal is not to become someone in particular but to remain fluid enough to learn from everything I encounter."*

You may modify this language to suit your own voice. What matters is the orientation it establishes: toward fluidity rather than fixation, toward learning rather than defending.

### Identity Fluidity Visualization (4 minutes)

Close your eyes and bring to mind three different perspectives you might inhabit today:

**First perspective (1 minute):** Imagine yourself as a complete beginner in your primary field of expertise. What would you notice that you currently overlook? What questions would you ask that you now consider too basic? Let yourself feel the freshness of not-knowing, the openness that comes before competence closes certain doors of perception.

**Second perspective (1 minute):** Imagine yourself as someone who will learn something important from the person you find most difficult or least impressive. Who might that be today? What might they know that you don't? This is not about false humility but about genuine recognition that every person has navigated experiences you haven't.

**Third perspective (1 minute):** Imagine yourself at the end of today, having been surprised by something you were certain about. What belief might be overturned? What assumption might prove unfounded? Allow yourself to feel the lightness of holding your certainties loosely.

**Integration (1 minute):** Notice that you have just occupied three different identity positions in your imagination, and that you remain yourself throughout all of them. This is the fluidity the practice cultivates—not losing yourself, but not being trapped either.

### Intention Setting (3 minutes)

Identify one specific domain you will enter today where you are not an expert. This might be:

• A conversation with someone from a different professional background

• A task you normally delegate or avoid

• A book, podcast, or article outside your usual interests

• A physical activity you haven't tried

• A question you will ask someone about their area of expertise

Write this intention down. The act of writing creates commitment and makes the intention specific enough to actually accomplish.

## Component 2: Throughout-Day Application

The morning practice establishes orientation; the throughout-day application is where liberation actually develops. This component requires no additional time—it simply changes how you engage with encounters that would happen anyway.

### The Three-Step Encounter Protocol

For each significant interaction during your day, move through these three steps:

**Step 1 — Assess the Position Dynamic:** Notice how you are instinctively positioning yourself relative to the other person. Are you in 'expert mode,' subtly or overtly establishing superiority? Are you in 'student mode,' perhaps too quickly deferring? Are you positioning as 'equal,' which can sometimes be its own form of subtle superiority? There is no wrong answer here—the point is simply to notice what is happening.

**Step 2 — Apply Position Fluidity:** Having noticed your default position, experiment with shifting it slightly. If you noticed yourself in expert mode, ask a genuine question about something you don't know. If you noticed excessive deference, offer one piece of your own knowledge or perspective. If you noticed a kind of competitive equality, try simply witnessing the other person without comparing. This is not about performing a different role but about loosening the grip of the default role.

**Step 3 — Extract the Learning:** After the encounter, take five seconds to identify one thing you learned. This might be factual information, but it might also be something about yourself, about human interaction, or about the limits of your previous understanding. If you genuinely cannot identify anything learned, that itself is important data—it may indicate that position-fixation prevented learning from occurring.

### Working with Hierarchy

Hierarchical relationships—with supervisors, subordinates, teachers, students—provide especially rich opportunities for liberation practice because the position assignments are explicit and socially reinforced.

**When you are in the 'higher' position:** Look for moments to genuinely learn from those below you in the hierarchy. This is not about false modesty or pretending not to know things you know. It is about recognizing that hierarchical position in one domain does not confer comprehensive superiority. The junior employee may understand customer experience better than you do. The student may see flaws in your argument that colleagues have been too polite to mention. Create conditions where this knowledge can flow upward.

**When you are in the 'lower' position:** Maintain respect for legitimate expertise while not abandoning your own capacity for critical assessment. You can learn from an expert while noticing where their expertise has limits. You can follow reasonable instructions while remaining alert to when those instructions might benefit from modification. The goal is neither rebellion nor submission but fluid engagement.

## Component 3: Achievement Transcendence Protocol

This component activates whenever you experience success, recognition, or achievement of any scale. The protocol is designed to prevent the natural human tendency to crystallize identity around accomplishment.

### The Three-Phase Response

**Phase 1 — Acknowledge (5 seconds):** Allow yourself to fully feel the satisfaction of achievement. This is not about suppressing positive emotion or pretending accomplishments don't matter. They do matter. The feeling of satisfaction is legitimate and healthy. Take five seconds to actually experience it rather than immediately deflecting or minimizing.

**Phase 2 — Identify Greater Challenge (60 seconds):** Within one minute of the acknowledgment, identify a challenge that is genuinely larger or more difficult than what you just accomplished. This should not be arbitrary or artificial but should represent an authentic next horizon. If you published a paper, what book would be harder? If you closed a deal, what systemic change would be more significant? If you helped one person, how might you help many? The point is not to diminish the current achievement but to place it in a larger context that prevents fixation.

**Phase 3 — Initiate Pursuit (within 24 hours):** Take at least one concrete action toward the greater challenge within 24 hours. This might be as simple as writing down a plan, sending an email, or doing ten minutes of research. The action need not be large, but it must be real. This prevents the achievement from becoming an endpoint and transforms it into a waypoint.

### Working with Recognition and Praise

When others praise you or offer recognition, there is a particular temptation to solidify identity around their positive view. The following guidelines help maintain fluidity:

Accept praise graciously. Deflecting or minimizing often comes from a position just as fixed as inflated pride—the position of 'humble person.' A simple 'thank you' is usually sufficient.

Do not reference the recognition in subsequent unrelated conversations. If you won an award and find yourself mentioning it when discussing unrelated topics, notice this as evidence of identity-fixation in progress.

Share credit authentically. Not as a performance of humility, but out of genuine recognition that most achievements involve contributions from others that often go unacknowledged.

## Component 4: Evening Integration Practice (15 minutes)

### Daily Liberation Coefficient Calculation (5 minutes)

Using the worksheet provided in Supplementary Material S2, calculate your daily Liberation Coefficient. This involves counting:

• Number of new domains entered (even briefly)

• Number of times you demonstrated position fluidity

• Number of times you referenced past achievements

• Number of position-fixation incidents you noticed

The calculation is simple: (New Domains + Fluidity Instances) divided by (Achievement References + Fixation Incidents + 1). The '+1' in the denominator prevents division by zero on particularly fluid days.

Do not become fixated on improving your score. The number is a mirror, not a grade. Some days will be lower than others due to circumstances outside your control. The practice is in the noticing, not in achieving high numbers.

### Critical Incident Review (5 minutes)

Identify one or two moments from the day when you noticed position-fixation occurring—either your own or someone else's. For each incident, briefly note:

• What triggered the fixation?

• What position was being defended or solidified?

• What was at stake (or seemed to be at stake)?

• What might fluidity have looked like in that moment?

This review is not about self-criticism. Position-fixation is deeply human and often unconscious. The review simply builds awareness, which over time creates more choice.

### Tomorrow Planning (3 minutes)

Identify tomorrow's domain expansion intention. What unfamiliar territory will you enter, even briefly? Write it down specifically enough that you will know whether you did it.

### Observer Visualization (2 minutes)

Close your eyes and replay the day as if watching someone else—with compassion but without identification. Notice how 'you' moved through various positions and situations. Notice moments of fluidity and moments of fixation with equal interest. The purpose is to cultivate the witnessing awareness that is itself never fixed in any position it observes.

## Troubleshooting Common Difficulties

**"I don't have time for this."** The morning and evening practices total 25 minutes. The throughout-day component requires no additional time—only a different quality of attention to interactions that would occur anyway. If 25 minutes is genuinely unavailable, begin with a 5-minute morning practice only and expand as the habit stabilizes.

**"I keep forgetting the throughout-day practice."** Set three phone alarms for random times during your workday. When an alarm sounds, pause for ten seconds and assess: what position am I currently in? This interrupt-based reminder helps build the habit until it becomes automatic.

**"My score isn't improving."** First, check that you are not becoming fixated on score improvement—this would be ironic but common. Second, remember that LC naturally fluctuates based on circumstances. A demanding week at work may reduce domain expansion opportunities. What matters is the long-term trend and, more importantly, the quality of awareness you bring to daily interactions.

**"This feels artificial."** All practices feel artificial at first. Walking felt artificial when you were learning it as a toddler. The artificiality gradually dissolves as the practice becomes habitual. If it still feels artificial after several weeks, the practice may need adaptation to fit your particular life circumstances.

**"I'm getting worse, not better."** This often indicates progress rather than regression. As awareness increases, you notice more fixation that was previously unconscious. A temporary increase in noticed fixation incidents is a normal and positive stage of development.

# Supplementary Material S2: Liberation Coefficient Calculation Worksheets

## Understanding the Liberation Coefficient

The Liberation Coefficient (LC) provides a quantitative snapshot of the balance between identity fluidity and identity fixation in a given time period. It is not a measure of worth or spiritual advancement—it is simply a tool for self-observation that makes patterns visible over time.

The basic formula is:

**LC = (New Domains Entered + Position Fluidity Instances) / (Past Achievement References + Position Fixation Incidents + 1)**

An LC greater than 1.0 indicates that fluidity behaviors outnumber fixation behaviors. An LC below 1.0 indicates the reverse. Neither is inherently good or bad—the coefficient simply makes visible what might otherwise remain unconscious.

## Defining the Components

### New Domains Entered

A 'new domain' is any area of activity, knowledge, or experience where you are not already established as competent. Examples include:

• Having a substantive conversation about a topic outside your expertise

• Attempting a skill you have not practiced before

• Reading or engaging with material from an unfamiliar field

• Asking genuine questions in an area where you lack knowledge

• Visiting a place or engaging with a community unfamiliar to you

The key criterion is genuine unfamiliarity—not performing unfamiliarity while actually maintaining expert status. Attending a beginner yoga class counts if yoga is genuinely new to you; it does not count if you are an experienced practitioner pretending to be a beginner.

### Position Fluidity Instances

A 'position fluidity instance' occurs whenever you consciously shift from a fixed identity position to a more fluid one. Examples include:

• Genuinely learning something from someone you typically teach or supervise

• Asking for help in an area where you could have maintained the appearance of competence

• Acknowledging uncertainty or error without excessive self-criticism or defensiveness

• Taking a perspective genuinely different from your habitual viewpoint

• Responding to criticism with curiosity rather than defensiveness

### Past Achievement References

A 'past achievement reference' is any invocation of previous accomplishment to establish current identity or status. Examples include:

• Mentioning credentials, awards, or publications when not directly relevant

• Beginning sentences with 'In my experience...' as a way of establishing authority

• Comparing current situations favorably to past successes

• Using past accomplishments to deflect current challenges or criticisms

Note: Referencing past experience when genuinely relevant to helping someone else does not count. The criterion is whether the reference serves identity-maintenance or genuine communication.

### Position Fixation Incidents

A 'position fixation incident' is any moment when you notice identity solidifying around a particular position. Examples include:

• Feeling defensive when your expertise is questioned

• Dismissing information because of its source rather than its content

• Maintaining a position in an argument beyond the point where evidence supports it

• Feeling threatened by others' success in your domain

• Avoiding situations where you might appear incompetent

## Daily Tracking Worksheet

Copy or adapt the following format for daily use:

**Date: ________________**

**NEW DOMAINS ENTERED:**

1. ________________________________________________

2. ________________________________________________

3. ________________________________________________

**Total New Domains: _____**

**POSITION FLUIDITY INSTANCES:**

1. ________________________________________________

2. ________________________________________________

3. ________________________________________________

**Total Fluidity Instances: _____**

**PAST ACHIEVEMENT REFERENCES:**

1. ________________________________________________

2. ________________________________________________

**Total Achievement References: _____**

**POSITION FIXATION INCIDENTS:**

1. ________________________________________________

2. ________________________________________________

**Total Fixation Incidents: _____**

**CALCULATION:**

LC = (_____ + _____) / (_____ + _____ + 1) = _____

**REFLECTION:**

What pattern do I notice? ________________________________________________

Tomorrow's intention: ________________________________________________

## Weekly Summary Worksheet

At the end of each week, calculate your average LC and note patterns:

**Week of: ________________**

Monday LC: _____ Tuesday LC: _____ Wednesday LC: _____

Thursday LC: _____ Friday LC: _____ Saturday LC: _____

Sunday LC: _____

**Weekly Average: _____**

**Highest LC day: _____ What made it high? ________________________________**

**Lowest LC day: _____ What made it low? ________________________________**

**Most common fixation trigger: ________________________________**

**Most effective fluidity practice: ________________________________**

**Intention for next week: ________________________________**

# Supplementary Material S3: Behavioral Assessment Battery

## Overview and Administration Guidelines

This battery comprises six behavioral tests designed to assess liberation-related patterns through observable behavior rather than self-report. The battery is intended for research purposes and requires trained administrators who understand both the theoretical framework and the ethical considerations involved in assessment.

**Administration Setting:** Tests should be administered in a comfortable, private environment. Participants should be informed that they are being assessed but should not be given details about what specific behaviors are being measured, as this could influence responses.

**Timing:** The complete battery requires approximately 2-3 hours spread across multiple sessions over a 30-day period. Some tests require observation of behavior in naturalistic settings.

**Scoring:** Each test yields a score from 0 to 1. The composite Liberation Coefficient is calculated as the mean of all six test scores.

## Test 1: Achievement Response Test

### Purpose

This test measures how quickly an individual moves from achievement to new challenge-seeking, versus dwelling in or consolidating around the achievement.

### Procedure

Prior to the testing period, identify a significant achievement the participant has recently experienced or will experience during the testing window. This might be a publication, promotion, award, successful project completion, or similar accomplishment.

Beginning from the moment of achievement (or recognition of achievement), measure the time until the participant initiates pursuit of a challenge that is clearly larger or more difficult than the achieved goal. 'Initiation' is defined as taking at least one concrete action (not merely thinking about or planning).

### Scoring

• New challenge initiated within 24 hours: Score = 1.0

• New challenge initiated within 48 hours: Score = 0.8

• New challenge initiated within 7 days: Score = 0.6

• New challenge initiated within 14 days: Score = 0.4

• New challenge initiated within 30 days: Score = 0.2

• No new challenge initiated within 30 days: Score = 0.0

### Validity Considerations

The 'greater challenge' must be verified as genuinely more difficult or significant, not merely different. An external rater familiar with the participant's field should confirm this assessment.

## Test 2: Authority Negation Test

### Purpose

This test measures the degree to which an individual in a position of authority takes actions that would reduce or eliminate that authority, versus actions that consolidate or expand it.

### Procedure

Place the participant in a position of clear authority for a defined period. This might involve leading a small team, teaching a class, supervising a project, or any role where others defer to their judgment.

Over a 30-day period, document all actions taken that relate to maintaining, expanding, or reducing the authority position. Actions are categorized as:

**Authority-reducing (AR):** Training others to replace oneself, delegating decision-making power, creating systems that function without one's oversight, publicly crediting others for successes

**Authority-neutral (AN):** Routine actions that neither expand nor reduce authority position

**Authority-consolidating (AC):** Centralizing decisions, taking credit for group accomplishments, creating dependencies on one's presence or approval

### Scoring

Score = AR / (AR + AC + 1)

A score of 1.0 would indicate only authority-reducing actions with no consolidating ones. A score approaching 0 would indicate predominantly consolidating behavior.

## Test 3: Recognition Response Test

### Purpose

This test measures the tendency to reference or invoke past recognition as a way of establishing current identity or status.

### Procedure

Identify a significant recognition event in the participant's recent past (award, public acknowledgment, positive review, etc.). Beginning 48 hours after the recognition, monitor all communications (with participant consent) including emails, social media, and conversations (through self-report diary) for a 30-day period.

Count the number of times the recognition is referenced in contexts where it is not directly relevant to the communication purpose.

### Scoring

• Zero irrelevant references: Score = 1.0

• 1-2 irrelevant references: Score = 0.8

• 3-5 irrelevant references: Score = 0.6

• 6-10 irrelevant references: Score = 0.4

• 11-20 irrelevant references: Score = 0.2

• More than 20 irrelevant references: Score = 0.0

## Test 4: Expert Demonstration Test

### Purpose

This test assesses whether expertise is demonstrated through credentials and institutional markers versus natural phenomena and direct evidence.

### Procedure

Ask the participant to explain a concept from their area of expertise to someone unfamiliar with the field. Record the explanation (with consent) and analyze the content for reliance on:

**Credential markers (CM):** References to degrees, publications, years of experience, institutional affiliations, prestigious mentors, etc.

**Direct demonstrations (DD):** Examples from observable phenomena, analogies to common experience, live demonstrations, invitations to verify claims independently

### Scoring

Score = DD / (DD + CM + 1)

High scores indicate reliance on direct evidence and phenomena; low scores indicate reliance on institutional authority markers.

## Test 5: Failure Integration Test

### Purpose

This test measures the response to having one's expertise or judgment publicly questioned or refuted.

### Procedure

Present the participant with a substantive critique of their work or a domain-specific failure. This should be genuine and relevant, not artificial or trivial. The critique should come from a credible source.

Observe and record the response. Categorize behaviors as:

**Defensive (D):** Dismissing the critique, attacking the critic's credibility, deflecting to others' failures, minimizing the significance

**Neutral (N):** Acknowledging without engagement, neither defensive nor integrative

**Integrative (I):** Asking clarifying questions, expressing gratitude for the feedback, identifying specific changes to be made, requesting further critique

### Scoring

Score = I / (I + D + 1)

## Test 6: Hierarchy Maintenance Test

### Purpose

This test measures the tendency to maintain hierarchical position versus demonstrate position fluidity in interactions with those perceived as lower in status.

### Procedure

Observe interactions between the participant and individuals who would typically be perceived as lower in relevant hierarchies (junior colleagues, students, service workers, etc.). Code behaviors as:

**Hierarchy-maintaining (HM):** Speaking more than listening, interrupting, using titles or credentials, directing rather than discussing, dismissing input

**Position-fluid (PF):** Active listening, genuine questions, acknowledging others' expertise, collaborative problem-solving, learning from the interaction

### Scoring

Score = PF / (PF + HM + 1)

## Composite Score Calculation

The composite behavioral Liberation Coefficient is calculated as:

**LC_behavioral = (T1 + T2 + T3 + T4 + T5 + T6) / 6**

**Interpretation:**

• LC ≥ 0.8: High liberation — consistently demonstrates fluidity across contexts

• 0.5 ≤ LC < 0.8: Moderate liberation — shows fluidity in some contexts but fixation in others

• LC < 0.5: Low liberation — predominantly fixated patterns across contexts

# Supplementary Material S4: Neuroimaging Task Specifications

## Overview

This document specifies the functional magnetic resonance imaging (fMRI) protocols designed to identify neural correlates of liberation-related processing. The protocols target brain regions and networks theoretically associated with self-referential processing, cognitive flexibility, and perspective-taking.

Three main scanning sessions are proposed: baseline measures, a 30-day training interval with practice, and post-training measures. The pre-post design allows assessment of neural changes associated with liberation practice.

## Technical Parameters

### Scanner Specifications

• Minimum field strength: 3.0 Tesla

• Head coil: 32-channel or higher recommended

• Multiband acquisition capability preferred

### Functional Acquisition Parameters

• Sequence: Echo-planar imaging (EPI)

• TR (repetition time): 1000-2000 ms

• TE (echo time): 30 ms

• Flip angle: 70-90 degrees

• Voxel size: 2.5 × 2.5 × 2.5 mm isotropic

• Slice acquisition: Interleaved ascending

• Number of slices: Sufficient for whole-brain coverage (typically 60-72)

### Structural Acquisition Parameters

• Sequence: T1-weighted MPRAGE

• Voxel size: 1.0 × 1.0 × 1.0 mm isotropic

• Duration: Approximately 5-7 minutes

## Task 1: Resting State Connectivity (8 minutes)

### Purpose

This task measures intrinsic functional connectivity patterns, particularly within and between the Default Mode Network (DMN), the frontoparietal control network, and the salience network. Liberation is hypothesized to be associated with increased flexibility of network configurations.

### Procedure

Participants are instructed to rest quietly with their eyes open, fixating on a central crosshair. They are told not to think about anything in particular and not to fall asleep. The scan lasts 8 minutes. Eye tracking is employed to verify wakefulness.

### Analysis

Primary measures include: (1) within-network connectivity of the DMN, (2) between-network connectivity (DMN-frontoparietal, DMN-salience), and (3) dynamic connectivity measures assessing flexibility of network configurations over time.

## Task 2: Self-Reference Task (10 minutes)

### Purpose

This task assesses neural responses during self-referential processing. Liberation is hypothesized to be associated with reduced medial prefrontal cortex (mPFC) activation during self-reference and increased temporoparietal junction (TPJ) activation, indicating a shift from fixed self-representation toward flexible perspective-taking.

### Procedure

Participants view trait adjectives (e.g., 'intelligent,' 'anxious,' 'creative') and respond to one of three prompts:

**Self condition:** 'Does this word describe you?' (Yes/No response)

**Other condition:** 'Does this word describe [familiar other's name]?' (Yes/No response)

**Case condition (control):** 'Is this word in uppercase letters?' (Yes/No response)

Stimuli are presented for 3 seconds each with a 1-2 second jittered inter-stimulus interval. Each condition includes 40 trials, presented in a pseudo-randomized order.

### Regions of Interest

• Medial prefrontal cortex (mPFC) — self-referential processing

• Posterior cingulate cortex (PCC) — autobiographical memory, self-continuity

• Temporoparietal junction (TPJ) — perspective-taking, mentalizing

• Anterior cingulate cortex (ACC) — cognitive control, conflict monitoring

## Task 3: Achievement-Related Stimuli Task (12 minutes)

### Purpose

This task measures neural responses to stimuli related to the participant's achievements and expertise. Liberation is hypothesized to be associated with reduced reward-related activation (ventral striatum) and reduced defensive responses (amygdala) when viewing achievement-related stimuli.

### Procedure

Prior to scanning, researchers work with participants to identify achievement-related stimuli specific to their history. These might include images of awards, publications, professional settings, or symbolic representations of accomplishments.

During scanning, participants view:

**Personal achievement stimuli:** Images related to participant's own achievements

**Others' achievement stimuli:** Images related to achievements of others in participant's field

**Neutral stimuli:** Non-achievement-related images matched for visual complexity

Stimuli are presented for 4 seconds each with 2-4 second jittered intervals.

### Regions of Interest

• Ventral striatum — reward processing

• Amygdala — threat/defensive processing

• Insula — interoceptive awareness

• Dorsolateral prefrontal cortex (dlPFC) — cognitive control

## Neural Liberation Index (NLI) Calculation

The Neural Liberation Index is a composite measure calculated from the three tasks:

**NLI = f(DMN_flexibility, TPJ_activation, mPFC_reduction, Amygdala_reduction)**

Specifically:

• DMN_flexibility: Dynamic connectivity measure from resting state

• TPJ_activation: Beta weights from Self > Case contrast

• mPFC_reduction: Inverse of beta weights from Self > Other contrast

• Amygdala_reduction: Inverse of beta weights from Personal Achievement > Neutral contrast

Values are z-scored within the sample before combination. The expected correlation between NLI and behavioral LC is r > 0.6.

# Supplementary Material S5: Cross-Cultural Validation Protocol

## Rationale

The Liberation Mathematics framework claims cross-cultural applicability—that liberation, operationally defined as perpetual self-transcendence through position-fluidity, represents a human universal rather than a culture-specific construct. This claim requires systematic testing across diverse cultural contexts.

Cross-cultural validation serves multiple purposes: (1) testing whether the operational definitions translate meaningfully across languages and cultural concepts, (2) examining whether the behavioral markers are universally relevant or culturally specific, (3) investigating whether the developmental trajectories differ across cultural contexts, and (4) refining the framework based on cross-cultural data.

## Target Traditions and Populations

Validation should occur across at least five distinct cultural-contemplative contexts:

**1. Buddhist Traditions (Southeast Asian and East Asian)**

Sites: Thailand (Theravada), Japan (Zen), Tibet/Nepal (Vajrayana). These traditions have explicit concepts of liberation (nibbāna/nirvana) and well-developed contemplative training systems. Key questions include whether the LC framework captures what these traditions mean by liberation and whether traditional practitioners score differently on LC measures than non-practitioners.

**2. Hindu Traditions (South Asian)**

Sites: India (multiple regions representing different sampradāyas). Hindu traditions include diverse concepts of liberation—moksha, kaivalya, mukti—with different emphases across Advaita, Dvaita, and Yoga traditions. Validation should include practitioners from multiple traditions to assess whether LC captures common elements.

**3. Islamic Sufi Traditions (Middle Eastern and South Asian)**

Sites: Turkey, Iran, Pakistan, Morocco. Sufi traditions describe fanā (annihilation of ego) and baqā (subsistence in God) in terms that may parallel liberation concepts. Validation examines whether LC measures correlate with traditional assessments of spiritual development within these lineages.

**4. Christian Contemplative Traditions (Western)**

Sites: Europe and North America (Carmelite, Trappist, Orthodox hesychast communities). Christian mystical traditions describe transformative union, kenosis (self-emptying), and deification (theosis) in ways that may parallel liberation concepts. Validation examines translation of concepts across theistic and non-theistic frameworks.

**5. Indigenous Traditions (Various)**

Sites: To be determined in consultation with indigenous communities. Many indigenous traditions have concepts of ego-transcendence, interconnection, and identity-fluidity embedded in their worldviews. Validation must proceed with particular sensitivity to cultural protocols and avoid extractive research practices.

## Translation and Adaptation Protocol

### Phase 1: Conceptual Mapping

Before translating instruments, researchers must work with cultural consultants to map Liberation Mathematics concepts onto local terminology. This is not simple translation but conceptual negotiation. For example, 'position-fixation' may not have a direct equivalent in a language but may map onto local concepts of attachment, ego-clinging, or pride.

For each core concept (liberation, position-fixation, domain expansion, ego capability, fresh perception), researchers should:

• Identify local terms that capture similar meanings

• Document areas of overlap and divergence

• Identify concepts in the local tradition that have no Liberation Mathematics equivalent

• Develop operational definitions that are culturally appropriate

### Phase 2: Instrument Translation

Use the standard translation-back-translation procedure:

1. Initial translation by bilingual expert familiar with Liberation Mathematics

2. Back-translation by independent bilingual expert unfamiliar with original

3. Comparison of back-translation with original; revision of discrepancies

4. Review by committee including cultural consultants

5. Pilot testing with native speakers; further revision

### Phase 3: Behavioral Marker Adaptation

The six-test behavioral battery may require cultural adaptation. For example, 'authority negation' may manifest differently in hierarchical versus egalitarian cultures. In some contexts, overt authority-reducing behavior might be culturally inappropriate even for highly liberated individuals.

For each test, researchers should:

• Identify culturally appropriate behavioral indicators of the target construct

• Develop locally meaningful scenarios for testing

• Validate adapted measures with local experts

• Document adaptations for cross-site comparison

## Sampling Strategy

Within each cultural context, sampling should include:

**Traditional practitioners:** Individuals with at least 5 years of serious engagement with the local contemplative tradition. These participants provide data on whether LC captures what traditions mean by advanced spiritual development.

**Recognized teachers/masters:** Individuals acknowledged within their tradition as having attained significant spiritual development. Their LC scores provide criterion validity data.

**General population:** Non-practitioner adults matched to practitioners on age, education, and socioeconomic status. These participants provide baseline comparison data.

**Recommended sample sizes:** Minimum 50 per group (150 total) per cultural site, for adequate power in cross-group comparisons.

## Analysis Plan

### Within-Culture Analyses

For each cultural site:

• Compare LC scores across practitioner, teacher, and general population groups

• Correlate LC scores with traditional assessments of spiritual development where available

• Examine internal consistency of LC measures

• Assess construct validity through correlation with related measures

### Cross-Cultural Analyses

Across all sites:

• Test measurement invariance of LC across cultures

• Compare mean LC levels across cultures (controlling for practitioner status)

• Examine whether the relationship between LC and well-being measures is consistent across cultures

• Identify culture-specific and universal components of liberation

## Ethical Considerations

Cross-cultural research on spiritual concepts requires particular ethical sensitivity:

**Avoid extractive research:** Research should benefit local communities, not merely extract data for external purposes. This includes sharing findings in accessible formats, training local researchers, and ensuring communities have voice in how findings are interpreted and disseminated.

**Respect traditional knowledge:** Liberation Mathematics is one framework among many. Research should not position it as superior to traditional understandings but as a complementary approach that may or may not capture what traditions consider most important.

**Protect sacred knowledge:** Some aspects of traditional teachings may be esoteric or restricted. Researchers must respect these boundaries and not press for information that traditions consider inappropriate to share.

**Obtain appropriate approvals:** Beyond institutional review board approval, research should obtain endorsement from relevant traditional authorities where appropriate.

## Timeline

A complete cross-cultural validation study requires approximately 3-5 years:

• Year 1: Conceptual mapping and instrument translation at all sites

• Year 2: Pilot testing and adaptation refinement

• Years 3-4: Main data collection

• Year 5: Analysis, framework refinement, and dissemination

This timeline may be extended for sites requiring more extensive relationship-building before data collection can appropriately begin.

**— End of Supplementary Materials —**
